# Pyrimidine Salvage Enzymes Are Essential for *De Novo* Biosynthesis of Deoxypyrimidine Nucleotides in *Trypanosoma brucei*

**DOI:** 10.1371/journal.ppat.1006010

**Published:** 2016-11-07

**Authors:** Christopher Leija, Filipa Rijo-Ferreira, Lisa N. Kinch, Nick V. Grishin, Nicole Nischan, Jennifer J. Kohler, Zeping Hu, Margaret A. Phillips

**Affiliations:** 1 Department of Pharmacology University of Texas Southwestern Medical Center at Dallas, Dallas, Texas, United States of America; 2 Instituto de Medicina Molecular, Faculdade de Medicina, Universidade de Lisboa, Lisboa, Portugal; 3 Department of Neuroscience, University of Texas Southwestern Medical Center at Dallas, Dallas, Texas, United States of America; 4 Graduate Program in Areas of Basic and Applied Biology, Instituto de Ciências Biomédicas Abel Salazar, Universidade do Porto, Porto, Portugal; 5 Department of Biophysics, University of Texas Southwestern Medical Center at Dallas, Dallas, Texas, United States of America; 6 Department of Biochemistry, University of Texas Southwestern Medical Center at Dallas, Dallas, Texas, United States of America; 7 Children’s Medical Center Research Institute, University of Texas Southwestern Medical Center at Dallas, Dallas, Texas, United States of America; University of Dundee, UNITED KINGDOM

## Abstract

The human pathogenic parasite *Trypanosoma brucei* possess both *de novo* and salvage routes for the biosynthesis of pyrimidine nucleotides. Consequently, they do not require salvageable pyrimidines for growth. Thymidine kinase (TK) catalyzes the formation of dTMP and dUMP and is one of several salvage enzymes that appear redundant to the *de novo* pathway. Surprisingly, we show through analysis of *TK* conditional null and RNAi cells that TK is essential for growth and for infectivity in a mouse model, and that a catalytically active enzyme is required for its function. Unlike humans, *T*. *brucei* and all other kinetoplastids lack dCMP deaminase (DCTD), which provides an alternative route to dUMP formation. Ectopic expression of human DCTD resulted in full rescue of the RNAi growth phenotype and allowed for selection of viable *TK* null cells. Metabolite profiling by LC-MS/MS revealed a buildup of deoxypyrimidine nucleosides in TK depleted cells. Knockout of cytidine deaminase (CDA), which converts deoxycytidine to deoxyuridine led to thymidine/deoxyuridine auxotrophy. These unexpected results suggested that *T*. *brucei* encodes an unidentified 5'-nucleotidase that converts deoxypyrimidine nucleotides to their corresponding nucleosides, leading to their dead-end buildup in TK depleted cells at the expense of dTTP pools. Bioinformatics analysis identified several potential candidate genes that could encode 5’-nucleotidase activity including an HD-domain protein that we show catalyzes dephosphorylation of deoxyribonucleotide 5’-monophosphates. We conclude that TK is essential for synthesis of thymine nucleotides regardless of whether the nucleoside precursors originate from the *de novo* pathway or through salvage. Reliance on TK in the absence of DCTD may be a shared vulnerability among trypanosomatids and may provide a unique opportunity to selectively target a diverse group of pathogenic single-celled eukaryotes with a single drug.

## Author Summary

Human pathogenic trypanosomatids are responsible for several life threatening diseases, together infecting 20 million people, while treatment is complicated by poor drug therapies. The unique biology of these organisms has led to the need for different drug therapies to be developed for each. Identification of enzymatic targets that could be used to develop a single drug capable of treating multiple parasites would be revolutionary. Herein we show that the trypanosomatid biosynthetic pathway used to synthesize key precursors for DNA biosynthesis is unexpectedly vulnerable. We find that seemingly redundant enzymes thymidine kinase and cytidine deaminase are required, not for their typical role in salvaging exogenous precursors, but are instead essential for *de novo* synthesis of thymine nucleotides. Trypanosomatids lack alternative routes to synthesize these nucleotides, which are found in other eukaryotic cells, while encoding a previously unknown activity that degrades them. For these reasons thymidine kinase is essential to support infection in mice and has strong potential as a new drug target. While our work focused on the causative agent of African sleeping sickness, the impact of our findings may extend to other pathogenic trypanosomatids and potentially to additional single-celled eukaryotic human pathogens.

## Introduction

The parasitic trypanosomatids are vector-borne single-celled eukaryotic pathogens that cause significant disease and mortality in tropical and subtropical countries [1]. Human African trypanosomiasis (HAT), Leishmaniasis and Chagas disease affect 20 million people combined, but control is hampered by lack of good drugs, drug resistance and challenges in drug administration [2, 3]. New drugs for the treatment of all three diseases are badly needed. HAT, also known as sleeping sickness, is caused by *Trypanosoma brucei*, an extracellular parasite that replicates in the blood in early stages of infection. It crosses the blood-brain barrier in later stages leading to progressive neurological complications that disrupt the sleep/wake cycle and which eventually progress to coma and death [4]. The WHO estimates that yearly cases have dropped below 10,000 [5]; however, attempts to fully eliminate the disease face the difficulties of a substantial animal reservoir and problematic drug therapies that have high toxicity or are difficult to administer in a rural setting.

**Fig 1:**
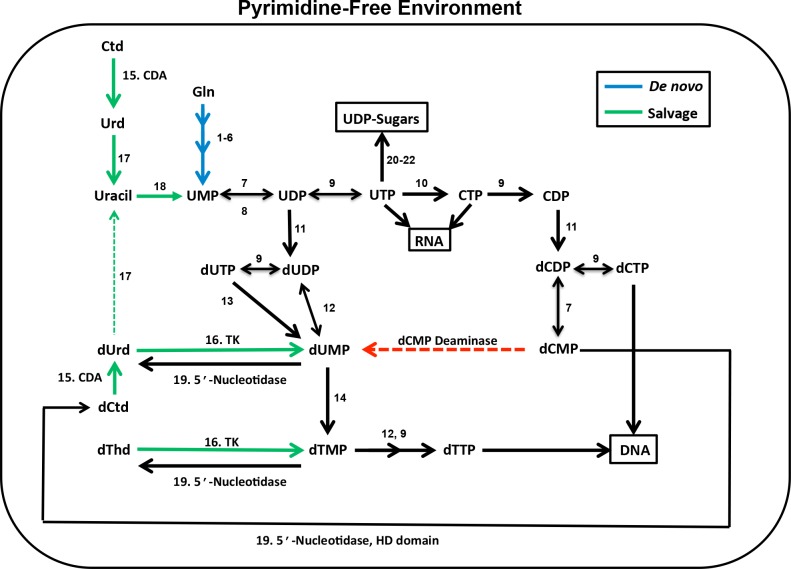
*T*. *brucei* pyrimidine pathway. Green lines salvage routes, blue lines *de novo* pathway, black lines interconversion routes, and the red dotted line indicates a reaction that is not present in trypanosomatids. The numbers above each arrow represent the enzyme catalyzing the reaction (EC number): **1–6**: carbamoyl phosphate synthase (6.3.5.5), aspartate carbamoyl transferase (2.1.3.2), dihydroorotase (3.5.2.3), dihydroorotate dehydrogenase (1.3.98.1), orotate phosphoribosyltransferase (2.4.2.10), orotidine 5-phosphate decarboxylase (4.1.1.23); **7** UMP-CMP kinase (2.7.4.14); **8**: nucleoside diphosphatase (3.6.1.6); **9**: nucleoside diphosphate kinase (2.7.4.6); **10**: cytidine triphosphate synthase (6.3.4.2); **11**: ribonucleoside diphosphate reductase (1.17.4.1); **12**: thymidylate kinase (2.7.4.9); **13**: deoxyuridine triphosphatase (dUTPase) (3.6.1.23); **14**: dihydrofolate reductase-thymidylate synthase (2.1.1.45); **15**:cytidine deaminase (CDA) (3.5.4.5); **16**: thymidine kinase (TK)(2.7.1.21); **17**: uridine phosphorylase (2.4.2.3); **18**:uracil phosphoribosyltransferase (2.4.2.9); 19: HD-domain 5’-nucleotidase (3.1.3.89); **20**: UDP-glucose pyrophosphorylase (2.7.7.9); **21**: UTP N-acetyl-α-D-glucosamine-1-phosphate uridylyltransferase (2.7.7.23); **22**: UDP-glucose 4-epimerase (5.1.3.2). The pathway was constructed based on the annotation described in [10] and modified to incorporate results from our studies. Additionally enzyme **7** was added based on the published report that one of seven encoded adenylate kinases (ADKG) was biochemically characterized and shown to be a UMP-CMP kinase [16].

Pyrimidine and purine biosynthesis is essential in trypanosomatids to generate precursors needed for the biosynthesis of DNA, RNA and sugar nucleotides [6, 7]. Purines are obtained entirely by salvage routes through an array of interconnected and seemingly redundant pathways [8]. However, despite this redundancy, enzymes from the purine pathway including GMP synthase have been shown to be essential for pathogenicity *in vivo* [9]. In contrast, trypanosomatids are able to synthesize pyrimidines either through the *de novo* biosynthetic pathway or through salvage of preformed nucleosides and bases [7, 10, 11]. Genes have been identified for the complete *de novo* pyrimidine biosynthetic pathway, for several key salvage enzymes and for a number of interconversion enzymes [12](Fig 1). Genetic knockout studies have shown that loss of various *de novo* pyrimidine biosynthetic enzymes leads to pyrimidine auxotrophy that can be rescued by exogenous uracil [13–15]. These findings are consistent with reports that uracil transport is the primary route for pyrimidine salvage [10]. However knockout of UMP synthase lead to avirulence in mice suggesting that *in vivo* pyrimidine salvage may be insufficient to completely overcome loss of the *de novo* pathway [15]. These studies have shown that despite apparent redundancy, enzymes in both the pyrimidine and purine biosynthetic pathways can be essential, especially for *in vivo* virulence of *T*. *brucei*, which as an extracellular parasite lacks access to high intracellular concentrations of metabolites.

*T*. *brucei* lack several transporters and enzymes found in higher eukaryotes that may make them more vulnerable to disruption of the pyrimidine biosynthetic pathway. The primary pyrimidine transporter in *T*. *brucei* preferentially takes up uracil, whereas transport of uridine, 2’-deoxyuridine, thymidine and cytidine is either non-existent or inefficient requiring high nucleoside concentrations [10]. Trypanosomatids lack dCMP deaminase (DCTD), an important contributor to dTTP biosynthesis through deamination of dCMP to dUMP in many higher eukaryotes [17, 18]. Instead trypanosomatids were thought to rely on dUTPase to convert uracil nucleotides synthesized by the *de novo* pathway into the thymine nucleotide pools [19].

*T*. *brucei* encodes three pyrimidine salvage enzymes: uracil phosphoribosyltransferase (UPRT), thymidine kinase (TK), and uridine phosphorylase (UPP), and additionally a cytidine deaminase (CDA) that can convert deoxycytidine to deoxyuridine (Fig 1). *T*. *brucei* UPP was shown to prefer uridine and deoxyuridine as substrates, and the enzyme was reported not to be essential based on RNAi knockdown studies [20]. In contrast to mammalian cells, which encode both cytosolic TK1 and mitochondrial TK2 [21–23], trypanosomatids possess only TK1. *Tb*TK is however a unique fusion of two TK domains that function as a pseudodimer. The N-terminal domain is catalytically inactive, while the C-terminal domain exhibits canonical TK activity [24]. Recent RNAi studies have suggested that TK is essential but a mechanistic understanding for why TK would be required has not emerged [25, 26].

Herein we report the results of genetic studies to determine the roles of pyrimidine salvage enzymes in pyrimidine nucleotide biosynthesis, growth and virulence in *T*. *brucei*. We found that knockdown of either *TK* or *CDA* led to cell death despite the finding that *T*. *brucei* does not require exogenous pyrimidines for growth. The *TK* conditional null mutant (c-null) could not be rescued by exogenous pyrimidines, whereas the *CDA* null was auxotrophic for thymidine or deoxyuridine. We were able to abolish the parasite’s reliance on TK by providing an alternative metabolic route to dUMP formation via DCTD. Metabolomic analysis showed that a significant cellular response to TK depletion was dead-end formation of pyrimidine nucleosides resulting in depletion of dTTP. These data taken together with the phenotype of the *CDA* null suggested the presence of an unidentified 5'-nucleotidase that promotes interconversion between the cytosine and thymine deoxynucleotide pools. Bioinformatics analysis suggested that *T*. *brucei* encodes a number of potential 5’-nucleotidases and we show that an HD-domain protein related to a well-characterized bacterial 5’-nucleotidase is able to catalyze this reaction. Thus our data show that TK is essential for the *de novo* biosynthesis of dTTP and that together with CDA and the 5’-nucelotidase these enzymes play a key role in maintaining balanced levels of the various deoxyribonucleotide pools. We propose that the absence of DCTD in trypanosomes, and other protists, may be a shared vulnerability, presenting a potential opportunity to develop a pan-trypanosomatid TK inhibitor to exploit this unique feature of the protozoan pyrimidine pathway.

## Results

### Thymidine kinase (TK) is essential for growth of bloodstream form (BSF) *T*. *brucei*

**Fig 2:**
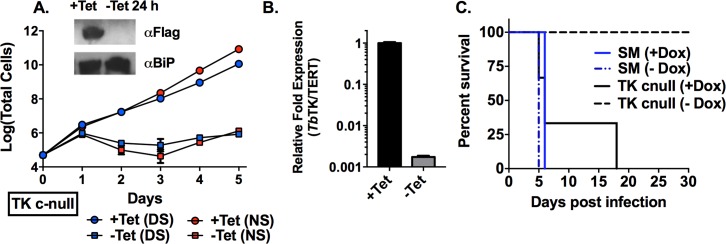
TK is essential for *in vitro* growth and infectivity in mice. A. Growth analysis of *TK* c-null cells and wild-type SM cells ±Tet. Expression of ectopic FLAG-tagged *Tb*TK is under Tet control, thus removal of Tet leads to loss of *Tb*TK expression. Cells were grown in HMI-19 medium supplemented with either normal serum (NS) or dialyzed serum (DS). Cell growth was monitored for the indicated days. Error bars represent standard deviation (SD) for triplicate biological replicates. Inset shows western blot analysis of FLAG-tagged *Tb*TK expression ±Tet for 24h. *Tb*BiP was detected as a loading control. B. qPCR analysis comparing mRNA expression levels of *Tb*TK to the TERT control ±Tet for 24 h. Error bars represent standard error of the mean (SEM) for triplicate data. C. Survival analysis of wild-type SM and TK c-null infected mice (±Dox) 1–30 days post infection for three mice per group.

To evaluate the essentiality of TK, we attempted to generate a *TK* null cell line in *T*. *brucei* BSF single marker (SM) cells. *T*. *brucei* is diploid, therefore minimally two alleles are present for each gene. Allelic replacement via homologous recombination was achieved by transfecting parasites with a PCR product containing a resistance marker flanked by the *TK* 5’ and 3’ UTRs. Removal of the first allele was successful; however we were unable to replace the last allele after repeated attempts, suggesting essentiality. To further evaluate this hypothesis, a *TK* conditional null (*TK* c-null) cell line was generated. The single allele knockout cells (SKO) were transfected with a vector conferring tetracycline (Tet) regulated expression of N-terminally FLAG tagged *T*. *brucei* TK (FLAG-*Tb*TK). Expression of FLAG-*Tb*TK was induced by addition of Tet and the remaining *TK* allele was successfully removed generating the *TK* c-null cell line. PCR amplification of the TK locus confirmed replacement by the two selectable markers (S1 Fig). To determine the effects caused by the loss of TK expression on cell growth, Tet was removed from the medium, which led to rapid growth arrest and near total cell death by day 3 (Fig 2A). Coincident with this growth arrest, *TK* transcript (by qPCR) and the TK protein (western blot) were depleted within 24 h after Tet removal, confirming good regulatory control of the ectopic *TK* copy (Fig 2A and 2B). Parasites reemerged several days later, likely due to the loss of Tet regulation. This has been reported to be a common phenomenon in *T*. *brucei*, likely due to mutations resulting in the loss of Tet regulation, e.g. [9, 27–29]. The *TK* c-null cells grew normally in pyrimidine-free medium (containing dialyzed fetal bovine serum) in the presence of Tet confirming that *T*. *brucei* is not auxotrophic for pyrimidines (Fig 2A). Upon subsequent removal of Tet, TK depletion led to cell death with a similar time course to medium containing non-dialyzed (normal) serum.

### TK depleted cells are unable to establish an infection in mice

*In vivo* studies were performed to determine if TK was essential to support *T*. *brucei* infection in mice. In parallel to *T*. *brucei* SM infected mice, two groups (n = 3) of *TK* c-null infected mice were given either doxycycline (Dox) treated water or water only. As expected mice infected with SM cells in either condition (+/- Dox) had detectable levels of parasitemia by 72 h post infection, with fatalities occurring in all mice in both groups by day 6 (Fig 2C and S2 Fig). Similarly, all *TK* c-null infected mice treated with Dox to maintain expression of the Tet-regulated TK copy eventually died within the timeframe of the study. One mouse in this group showed a delayed time before succumbing to parasitemia suggesting some variability in TK expression levels in the *TK* c-null cells. Mice infected with the *TK* c-null strain treated only with water remained healthy and had no detectable levels of parasitemia past 30 days. Thus we conclude that TK is essential for *T*. *brucei* virulence and infectivity *in vivo*.

### TK RNAi-induced growth arrest is reversed by expression of TK from multiple species

The finding that TK is essential in *T*. *brucei* is puzzling as no clear mechanistic role for TK in parasite fitness is apparent. *T*. *brucei* requires TK, which is a pyrimidine salvage enzyme; yet there is no requirement for salvageable pyrimidines for growth. To gain further mechanistic insight into this conundrum, we sought to address three possible explanations for the essentiality of TK: 1) parasites require an active TK enzyme, but it makes a novel product; 2) the TK protein, but not its catalytic activity is needed in some regulatory capacity; 3) parasites require formation of dUMP/dTMP by TK to balance pathway flux even under conditions where all pyrimidine precursors originate from the *de novo* pathway.

**Fig 3:**
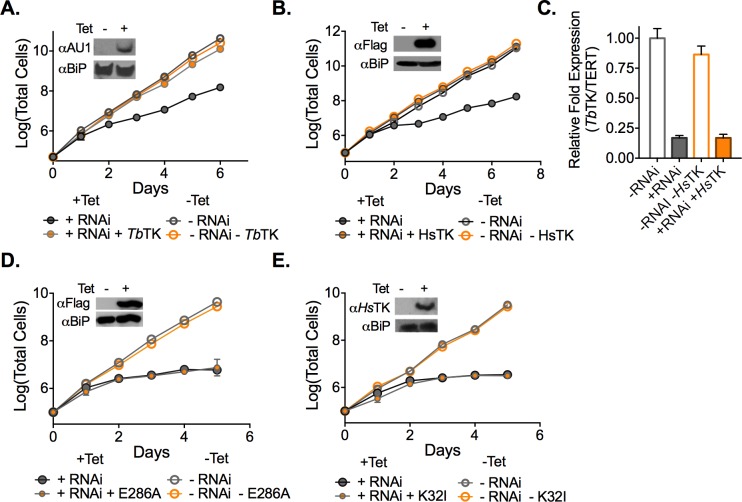
Catalytically active TK is required to rescue the *TK* RNAi growth phenotype. A-B. Growth analysis of TK RNAi cells (±Tet) expressing *Tb*TK or *Hs*TK under Tet control. Cell growth was monitored for the indicated days. Error bars represent SD for triplicate biological replicates. C. qPCR analysis of *Tb*TK mRNA levels in *TK* RNAi knockdown cells in the absence and presence of the *Hs*TK rescue plasmid 48 h after Tet addition. Error bars represent SEM for triplicate data. Data were normalized to TK levels in wild-type SM cells. D-E. Growth analysis of *TK* RNAi cells (±Tet) expressing active-site mutant TK enzymes, *Tb*TK E286A or *Hs*TK K32I under Tet control. Error bars represent SD for triplicate biological replicates. Insets show western blots of the AU1-*Tb*TK (A), FLAG-*Tb*TK (D) or FLAG-*Hs*TK (B,E) rescued RNAi lines comparing ±Tet for 48 h, though in panel E, *Hs*TK K32I was detected with a *Hs*TK antibody. *Tb*BiP was detected as a loading control.

To provide additional mechanistic insight, an inducible RNAi cell line targeting *TK* mRNA was created so that we could easily introduce various rescue plasmids to address our mechanistic hypotheses. A Tet-regulated vector capable of producing a hairpin transcript targeting the 3’UTR of *TK* was generated and transfected into the *TK* SKO cell line. Induction of *TK* RNAi by addition of Tet led to a significant growth defect, although the growth defect was not as severe as observed for *TK* c-null cells (Fig 3A and 3B). *TK* mRNA expression was reduced to 20–25% of wild-type control levels by RNAi targeting the *TK* transcript (Fig 3C). However, the reduction in *TK* transcript levels was less in comparison to that observed in the *TK* c-null cells, explaining why the effect on cell growth was less pronounced.

To shed light on whether a novel TK product was being formed we transfected parasites with plasmids encoding rescue proteins from three different sources: AU1-tagged *T*. *brucei* TK, FLAG-tagged human *Hs*TK, and *Herpes simplex* TK (*Hsv*TK). *Hs*TK has been shown to have more stringent substrate specificity than the *T*. *brucei* enzyme [24], whereas the viral *Hsv*TK possesses broader substrate specificity than the human enzyme [30]. Rescue protein expression was also under control of Tet promoter. Thus, addition of Tet to these cells induces simultaneous knockdown of endogenous *TK* and expression of the tagged rescue protein. We found that the growth phenotype was reversed by expression of TK from all three species: *T*. *brucei* (*Tb*TK)(Fig 3A), human (*Hs*TK) (Fig 3B) and viral TK (*Hsv*TK) (S3 Fig). Expression of *Tb*TK and *Hs*TK was confirmed by western blot (Fig 3A and 3B) and knockdown of endogenous *TK* was monitored by qPCR (Fig 3C). Viral TK expression was confirmed by the observance of ganciclovir sensitivity that was less apparent in cells expressing *T*. *brucei* TK (S3 Fig). Ganciclovir is a subversive substrate of *Hsv*TK leading to premature chain termination of newly synthesized DNA [31]. The ability of both *Hs*TK and *Hsv*TK to rescue the *TK* RNAi growth phenotype shows that *T*. *brucei* TK is unlikely to catalyze a novel reaction, as the required activity is present in enzymes from other species that are known to have a range of substrate specificities.

### Catalytically active TK is required for rescue of *Tb*TK RNAi cells

To confirm that *Tb*TK’s essential function is dependent on catalytic activity, mutations in the active site of both *Tb*TK and *Hs*TK were created. We targeted two conserved residues (S4 Fig) with described roles in the TK catalytic mechanism: *T*. *brucei* E286, which is reported to function as a proton acceptor [32] and human K32 which is an essential ATP binding residue [33]. Rescue plasmids were constructed as described above with the mutant TKs under the control of the Tet promoter and transfected into the *TK* RNAi line. In contrast to the wild type enzymes, neither the *Tb*TK E286A nor *Hs*TK K32I active site mutants were able to reverse the RNAi induced growth phenotype (Fig 3D and 3E). These data demonstrate that TK catalytic activity is required for its role in *T*. *brucei* cell survival.

### Deoxyuridine supplementation partially rescues the RNAi-induced growth phenotype

**Fig 4:**
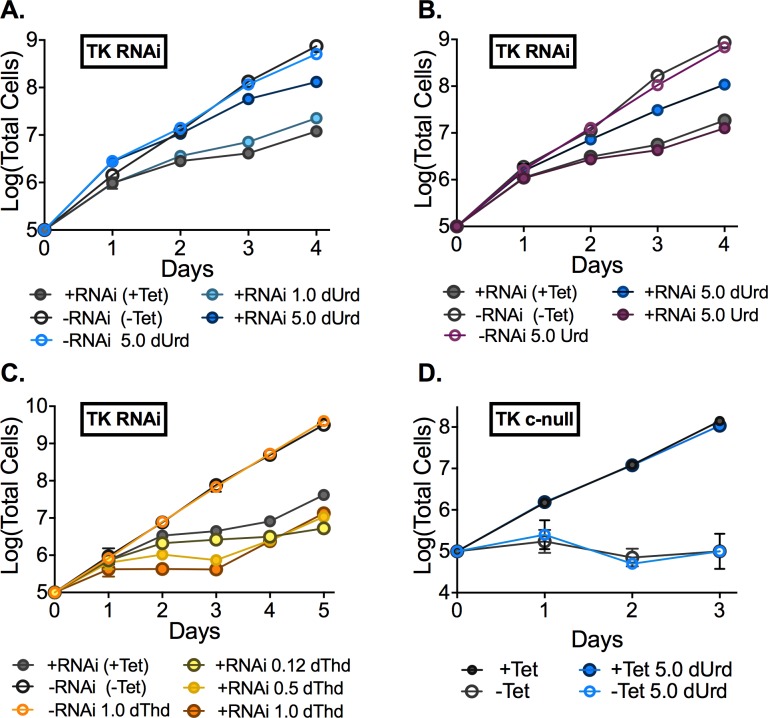
Effects of nucleoside supplementation on growth in *TK* RNAi or c-null cells. Growth analysis of *TK* RNAi cells supplemented with: A. dUrd (1 or 5 mM). B. Urd and dUrd (5 mM). C. dThd (0.5 or 1 mM) D. dUrd supplementation of *TK* c-null cells (±Tet). *TK* c-null cells express an ectopic copy of *Tb*TK under Tet control. Error bars represent SD for triplicate biological replicates.

While our data clearly show that *T*. *brucei* is not a pyrimidine auxotroph, we exploited the fact that the *TK* RNAi line retains partial TK activity (the knockdown is only 75–80% effective by RNAi (Fig 3C)) to assess whether we could use pyrimidine rescue to determine which TK product was needed for *T*. *brucei* growth. We found that high concentrations (significantly above physiological levels) of deoxyuridine (dUrd) resulted in the partial rescue of the RNAi growth phenotype (Fig 4A and S5A Fig) (5 mM rescued but 1 mM did not). However, similar levels of uridine (Urd) (Fig 4B and S5B Fig) or thymidine (dThd)(Fig 4C and S5C Fig) did not restore growth and dThd (0.15–1.0 mM) was in fact growth inhibitory to the *TK* RNAi +Tet induced cells but not to cells that expressed TK (-Tet), suggesting some type of feedback regulation. In contrast, the addition of dUrd or uracil to *TK* c-null cells, which are >99% depleted of *Tb*TK, were unable to circumvent lethality of the *TK* knockout showing that TK activity is required for dUrd rescue (Fig 4C and S6 Fig). These data confirm that TK plays an essential role in maintaining dUMP pools.

### Human dCMP deaminase rescues TK-deficient cells

**Fig 5:**
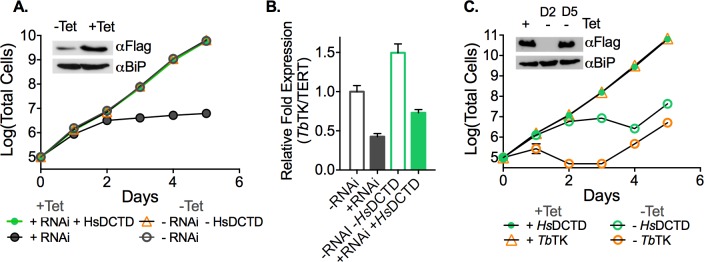
*Hs*DCTD rescues the growth defect in *Tb*TK RNAi and *Tb*TK null cell lines. A. Growth curves for *TK* RNAi cells or *TK* RNAi cells containing a Tet-regulated expression plasmid for *Hs*DCTD. Cell growth was monitored ±Tet for the indicated days. Error bars represent SD for triplicate biological replicates. Inset shows a Western blot of *Hs*DCTD expression ± Tet at 48 h. B. qPCR analysis of *Tb*TK mRNA expression in both the *TK* RNAi cell line and the *TK* RNAi *Hs*DCTD rescue line (±Tet 48 h). Error bars represent SEM for triplicate data. Data were normalized to the -Tet control, which is in the background of the single allele TK knockout. C. Growth analysis of *TK* null cells expressing either FLAG-tagged *Tb*TK (c-null) or FLAG-tagged *Hs*DCTD under the control of the Tet promoter. Error bars represent SD for triplicate biological replicates. Inset shows western blot analysis of the *Hs*DCTD *TK* null cells 2 days and 5 days after Tet withdraw.

The ability of dUrd to partially reverse the RNAi growth phenotype highlights an interesting feature in *T*. *brucei* pyrimidine metabolism. In most mammals, it has been suggested that a significant portion of dTTP is derived from dUMP produced by dCMP deaminase (DCTD)[17, 18]. Trypanosomatids lack this enzyme, restricting the number of metabolic routes dedicated to dUMP formation. We hypothesized that due to the lack of DCTD, trypanosomatids require TK to supplement dUMP pools. To test this hypothesis a Tet-regulated vector encoding human DCTD (*Hs*DCTD) was transfected into the *TK* RNAi cell line to drive simultaneous expression of *Hs*DCTD and knockdown of endogenous *Tb*TK. The expression of FLAG-tagged *Hs*DCTD completely rescued the *TK* RNAi-induced growth phenotype (Fig 5A). Expression of FLAG-tagged *Hs*DCTD was confirmed by western blot and qPCR analysis confirmed that *TK* mRNA expression was simultaneously reduced (Fig 5B). To further demonstrate that *Hs*DCTD can functionally replace TK, a *TK* null cell line was created in the background of FLAG-tagged *Hs*DCTD Tet-regulated expression plasmid. Both *TK* alleles were replaced by selectable markers through homologous recombination in the presence of Tet to maintain expression of *Hs*DCTD. PCR amplification of the region flanking the *TK* 5’ and 3’ UTRs confirmed replacement of *TK* with the selectable markers (S1 Fig). PCR analysis also confirmed that the *TK* gene was no longer detectable in genomic DNA from the *TK* null cells. Removal of Tet from this cell line led to depletion of FLAG-tagged *Hs*DCTD and resulted in a severe growth phenotype by day 2 after Tet removal (Fig 5C). Cell growth of the *Hs*DCTD *TK* null line was less severely impacted than the *TK* c-null line expressing Tet-regulated *Tb*TK from the rescue plasmid, perhaps reflecting a higher residual expression level of *Hs*DCTD (Fig 5C). By day 5 after Tet withdrawal cells began growing again coincident with re-expression of *Hs*DCTD, again suggestive of emergence of cells that have mutations leading to loss of Tet regulation (Fig 5C). Thus, the data support the hypothesis that TK is essential in *T*. *brucei* and that its role is to contribute to the formation of dUMP in the absence of DCTD.

### Metabolomic analysis reveals an accumulation of nucleoside substrates upon TK depletion

**Fig 6:**
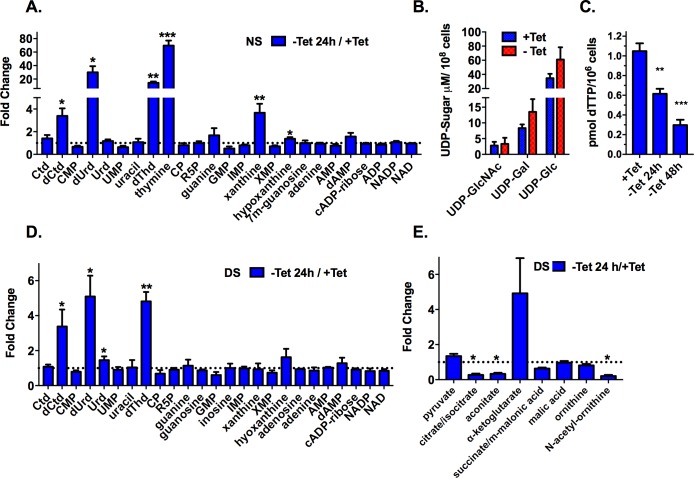
Metabolomic analysis of TK c-null cells. A. Detected pyrimidine and purine bases, nucleosides and nucleotides for cells grown in HMI-19 medium supplemented with normal serum (NS). The ratios (fold change) of metabolite levels in the absence of Tet for 24h compared to cells grown with Tet are plotted. B. HPAEC analysis of nucleotide sugars ±Tet at 24 h. C. Quantitation of dTTP by enzymatic assay ± Tet at 24 and 48 h for cell grown in HMI-19 supplemented with NS. D. Fold change of detected pyrimidine and purine bases, nucleosides and nucleotides ± Tet at 24 h for cells grown in HMI-19 medium supplemented with dialyzed serum (DS). E. Fold change of TCA intermediates ±Tet at 24 h for cells grown in HMI-19 medium supplemented with DS. Metabolites shown for C and D are from the same experiment. Data for additional detected metabolites for the normal serum (A) and dialyzed serum (D and E) studies are presented in Supplemental Figures. All data were collected in biological triplicate and error bars represent the SEM calculated for the ±Tet ratio by Graph Pad Prism using the baseline-correction algorithm. For A, D and E, multiple T test analysis was performed in GraphPad Prism comparing the +Tet and -Tet conditions for each study. Statistical significance was determined without correction for multiple comparisons and without assuming a consistent standard deviation. For C, data were analyzed using one way ANOVA with Dunnett’s multiple comparison test. Metabolites that showed a significant difference between the conditions are marked * P<0.05, ** P<0.01, *** P<0.001. Abbreviations are common nomenclature or have been previously defined except for CP, carbamoyl phosphate, R5P, ribose 5’-phosphate, 7m-guanosine, 7-methyl guanosine, succinate/m-malonic acid, succinate/methyl-malonic acid.

Analysis of ~130 soluble metabolites from *TK* c-null cells was performed by liquid chromatography-tandem mass spectrometry (LC-MS/MS) to determine the impact of TK depletion on metabolite pools (Fig 6 and S7–S9 Figs and S1 Appendix). Extracts were collected from *TK* c-null cells grown in medium containing normal non-dialyzed serum at 24 h ± Tet. An early time point was selected so that metabolite pools would be less affected by non-specific changes resulting from cell death later in the time course. Significant changes in the measured metabolite levels were mostly confined to pyrimidine nucleosides: there was a 15-30-fold accumulation of the TK substrates dUrd and dThd, a 3-fold accumulation of dCtd, and a 70-fold increase in thymine levels (Fig 6A). In contrast, the pyrimidine nucleotides CMP and UMP were not significantly changed by TK depletion, suggesting that the *de novo* pathway was able to maintain the uridine nucleotide pools. In further support, HPLC analysis of UDP-sugars was performed (Fig 6B). Nucleotide sugars are formed from UTP, thus their measurement provides a read-out of effects on intracellular UTP concentrations. The relative abundance of UDP-GlcNAc, UDP-Galactose, and UDP-Glucose were not significantly changed by TK depletion, confirming that UTP pools are not linked to TK activity. Thymidine nucleotide pools were not detected by the LC-MS/MS analysis so instead we quantitated dTTP levels using an enzymatic assay and found that dTTP levels were reduced to 30% of control levels 48 h after Tet removal, confirming TK is essential for synthesis of dTTP (Fig 6C).

To better understand the consequences of TK depletion in a pyrimidine-free medium the LC-MS/MS analysis was repeated for *TK* c-null cells grown in medium supplemented with dialyzed serum (Fig 6C and S7–S9 Figs and S1 Appendix). Similar to the results for cells grown in normal serum based medium, we observed a statistically significant buildup of TK substrates dUrd and dThd, and of the dUrd precursor dCyd, though the increases (3-6-fold) were less than observed for cells grown in normal serum based medium. We also observed changes in the levels of TCA intermediates including decreases in citrate and aconitate and an increase in α-ketoglutarate, and perturbations in other metabolites related to pyrimidine biosynthesis including decreased carbamoylphoshate (CP) and acetyl-ornithine (30 and 80%, respectively) and a 2-fold increase in homocysteine (Fig 6C and 6D). Not all of these latter changes reached statistical significance. Some modest effects were also observed on several purines: an increase in xanthine and/or hypoxanthine was observed in both medium conditions, which may suggest some type of cross-regulation between pyrimidine and purine biosynthetic pathways. Thymine was only observed in cells grown in medium supplemented with non-dialyzed serum, which could supply a source for thymine, confirming genomic analysis that suggests *T*. *brucei* does not encode thymine biosynthetic enzymes.

### Deletion of cytidine deaminase induces pyrimidine auxotrophy

**Fig 7:**
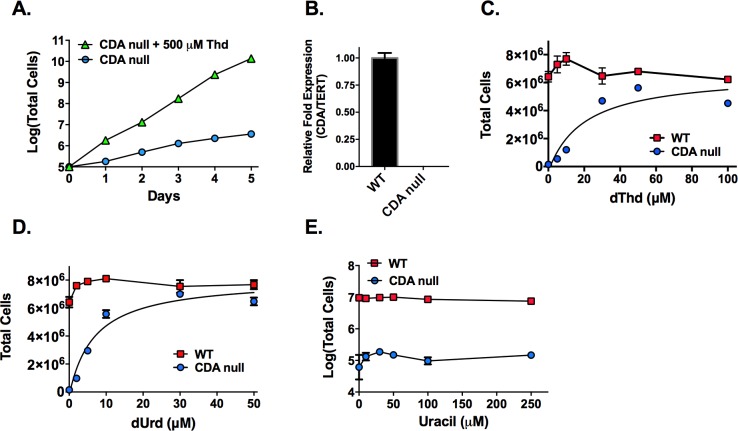
Deletion of the *T*. *brucei CDA* gene induces pyrimidine auxotrophy. A. Growth curves for *CDA* null cells grown in HMI-19 or HMI-19 media supplemented with 500 μM dThd. Cell growth was monitored for the indicated days. Error bars represent SD for triplicate biological replicates. B. qPCR analysis of CDA expression in wild-type SM and *CDA* null cells. Error bars represent SEM for triplicate data. C-E. Growth analysis of *CDA* null cells supplemented with dThd (C), dUrd (D) or uracil (E) over a range of concentrations 48 h post dThd withdrawl. Error bars represent the range for duplicate biological replicates. dThd and dUrd dose response curves were fitted to the Agonist vs response (three parameters) equation in GraphPad Prism (line represents the fit), to obtain ED_50_ for growth stimulation. ED_50_ = 20 μM (2.9–61) for dThd and 6.8 μM (3.8–13) for dUrd, where values in parenthesis represent the 95% confidence interval.

The finding that the deoxypyrimidine nucleoside pools buildup upon TK depletion for cells grown in pyrimidine-free (dialyzed serum based) medium suggests that an undiscovered biosynthetic route for formation of deoxypyrimidine nucleosides must be present in *T*. *brucei*. To investigate if deoxycytidine could be involved in formation of dUMP and dTMP we decided to characterize the effects of depleting cytidine deaminase (CDA) on parasite growth. Based on the current annotation of the pyrimidine biosynthetic pathway, CDA like TK should catalyze a redundant reaction, since the deoxynucleotide pools can be supplied from the *de novo* pathway. However, attempts to generate a *CDA* null were unsuccessful, and indeed the *CDA* null could only be obtained in the presence of 500 μM dThd (Fig 7 and S10 Fig). Upon removal of dThd, *CDA* null cells exhibited a severe growth defect (Fig 7A). Cells cultured for longer eventually died around day 7–10 after dThd removal. Additional pyrimidine rescue studies revealed that both dThd and dUrd were capable of rescuing growth (EC_50_ of 6–20 μM), whereas uracil (up to 250 μM) was not (Fig 7C–7E). The concentrations of dThd and dUrd required for rescue are significantly above reported human blood levels (range of 0.2–0.6 μM; http://www.hmdb.ca).

*CDA* null cells were grown ± dThd for 12 h in media supplemented with dialyzed FBS and the metabolite pools were analyzed by LC-MS/MS as described above. Overall the effects of CDA depletion on cellular metabolism were very similar to the effects observed after depletion of TK, confirming a link between the roles of the two enzymes (S11 Fig, S12 Fig and S1 Appendix). We observed a significant buildup (~7-fold) in the CDA substrate, dCtd, for the *CDA* null cells grown in the absence of Thd that was accompanied by changes in TCA intermediates and other metabolites that serve as precursors for *de novo* pyrimidine biosynthesis. These changes include increases in α-ketoglutarate (11-fold), glutamate (7-fold) and homocysteine (2-fold), and decreases in carbamoyl phosphate (2-fold). We also observed decreased levels of several amino acids, of polyamines, particularly N-acetyl putrescine and decreased levels of several purine mono-phosphate nucleotides. Neither dUrd nor dThd were detected in CDA null cells in the presence or absence of added dThd, suggesting they were either not formed (dUrd) or rapidly metabolized (dThd). In the absence of CDA, the buildup of dCtd suggests that the pyrimidine deoxynucleotides have been sequestered into a dead-end product that would be expected to lead to depletion of key nucleotides and to cell death as was observed for the TK null cells. These data support a role for CDA in the interconversion of deoxycytidine, deoxyuridine and thymidine pools, which is needed to balance these pools in a cell like *T*. *brucei* that expresses TK but not DCTD.

### *T*. *brucei* proteins with domains that can perform 5’-nucleotidase activity

In order to identify the potential enzyme(s) responsible for converting deoxypyrimidine nucleotides into their corresponding nucleosides we undertook a bioinformatics analysis of the *T*. *brucei* genome. 5'-nucleotidase activities that catalyze formation of deoxyuridine, deoxycytidine, or thymidine from their respective mono-phosphate nucleotides are ascribed to two EC numbers (EC3.1.3.5 and EC3.1.3.89). Inspection of domain types performing these activities revealed nine protein families defined by PFAM, with all having homologous structure representatives in the PDB (Table 1). The families further merge into five different homologous fold types; including three different α/β sandwich folds (phosphoglycerate mutase-like, SurE-like, and HAD domain-related), one α+β four-layer sandwich fold (metallo-dependent phosphatases), and one all-α fold (HD-domain). The phosphoglycerate mutase-like representatives are limited to mammalian acid phosphatases, prostate (ACPP) enzymes that convert extracellular AMP to adenosine (i.e. ecto 5' nucleotidase activity) [34]. An additional identified enzyme (NT5E) from the metallo-dependent phosphatase fold group exhibits a similar ecto 5'-nucleotidase activity [35]. Examples of this fold group in both eukaryotes and bacteria contain signal peptides and are extracellular. In contrast, the remaining SurE, HAD domain-related, and HD-domain representatives appear cytosolic.

**Table 1:** 5'-nucleotidase PFAM domain representatives.

Entry	Gene	EC	Pfam ID	PFAM Family	PDB ID	ECOD H
P15309	ACPP	3.1.3.5	PF00328	His_Phos_2	1rpa^1^	Phosphoglycerate mutase-like
P0A840	surE	3.1.3.5	PF01975	SurE	1l5x^1^	SurE-like/CoA-transfer-ase family III (CaiB/BaiF)
P21589	NT5E	3.1.3.5	PF00149; (PF02872)	Metallophos; (5_nucleotid_C)	1ush^1^	Metallo-dependent phosphatases
P49902	NT5C2	3.1.3.5	PF05761	5_nucleotid	2bde^1^	HAD domain-related
Q9H0P0	NT5C3A	3.1.3.5	PF05822	UMPH-1	2bdu^1^	HAD domain-related
Q9BXI3	NT5C1A	3.1.3.5	PF06189	5-nucleotidase	2b82^2^	HAD domain-related
P0AF24	nagD	3.1.3.5	PF13344	Hydrolase_6	5aes^1^	HAD domain-related
P0A8Y1	yjjG	3.1.3.5	PF13419	Hydrolase_2	4eek^1^	HAD domain-related
P76491	yfbR	3.1.3.89	PF13023	HD_3	3kh1^1^	HD-domain/PDEase-like

Representative ^1^PFAM family structure or ^2^representative homologous structure identified by HHPRED.

We found evidence for all of the 5'-nucleotidase homologous fold types in the *T*. *brucei* genome with the exception of the SurE fold class (S1 Table). We identified nine phosphoglycerate mutase-like sequences, 33 metallo-dependent phosphatase sequences, 16 HAD domain-related sequences, and one HD-domain sequence (S1 Table). The presence of numerous examples of potential 5'-nucleotidase domains, many of which are annotated as hypothetical proteins, suggests multiple possible proteins that *T*. *brucei* could use to form deoxyuridine, deoxycytidine, or thymidine *de novo*. However, three of the identified genes possess specific PFAM domains described as having 5’-nucleotidase activity (EC 3.1.3.5 or EC 3.1.3.89) and thus are the highest ranked candidates. One encodes a HD domain protein: hypothetical protein (Tb09.211.2190) and two encode HAD-like domains: a putative *p*-nitrophenylphosphatase (Tb927.8.7510) and a hypothetical protein (Tb09.211.1880). The *T*. *brucei* HD domain protein is a homolog of *E*. *coli* 5’-nucleotidase YfbR (S13 Fig) while the *T*. *brucei* HAD-domain proteins are related to enzymes shown to have 5’-nucleotidase activity in both yeast and *E*. *coli*. [36–38]. These enzymes have been reported to have broad substrate specificity functioning on all three pyrimidine deoxyribose monophosphates.

### *T*. *brucei* HD domain protein encodes a metal dependent 5’-nucleotidase

**Fig 8:**
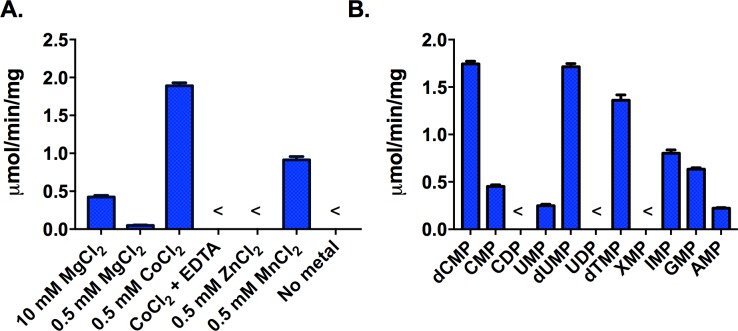
Steady-state kinetic analysis of *T*. *brucei* HD domain 5’-nucleotidase. A. Metal ion dependence. dCMP (1 mM) was used as the substrate and metal concentrations are noted on the figure. B. Substrate preference. Substrate concentrations were 1 mM and these assays were run in the presence of 0.5 mM Co^+2^. The < symbol on the graph indicates that the activity was below the level of detection. Data were collected in triplicate and error bars represent the SD of the mean.

To provide support for our hypothesis that *T*. *brucei* encodes a 5’-nucleotidase we cloned, expressed and purified the recombinant *T*. *brucei* HD domain homolog (Tb09.211.2190) of bacterial 5’-nucleotidase YfbR (S14 Fig). We found that the *T*. *brucei* YfbR-like HD protein showed a metal dependent 5’-nucleotidase activity (Fig 8A). Similar to the bacterial enzyme it was most active in the presence of Co^+2^ (0.5 mM), but activities within 2-4-fold of levels observed for Co^+2^ were also obtained using Mn^+2^ (0.5 mM) and physiological levels of Mg^+2^ (10 mM). No activity was observed in the presence of Zn^+2^ or EDTA. The specific activity of the *T*. *brucei* HD domain 5’-nucleotidase was very similar to the reported activity of *E*. *coli* YfbR [38]. The *T*. *brucei* enzyme showed a broad substrate range functioning on both pyrimidine and purine deoxyribonucleoside and ribonucleoside 5’-monophosphates, though it was most active on the deoxypyrimidine nucleotides (dCMP, dUMP and dTMP)(Fig 8B). It showed no activity towards diphosphate nucleotides. The *T*. *brucei* enzyme was somewhat more promiscuous then *E*. *coli* YfbR, which was unable to catalyze hydrolysis of ribonucleoside 5-monophosphates [38]. Both *T*. *cruzi* and *Leishmania* encode homologs of the *T*. *brucei* HD-domain 5’-nucleotidase (S1 Table and S13 Fig) suggesting they both also will be able to convert 5’-deoxyribonucleotide monophosphates to their respective nucleosides.

### Single-celled eukaryotic pathogens that encode TK but lack DCTD

**Fig 9:**
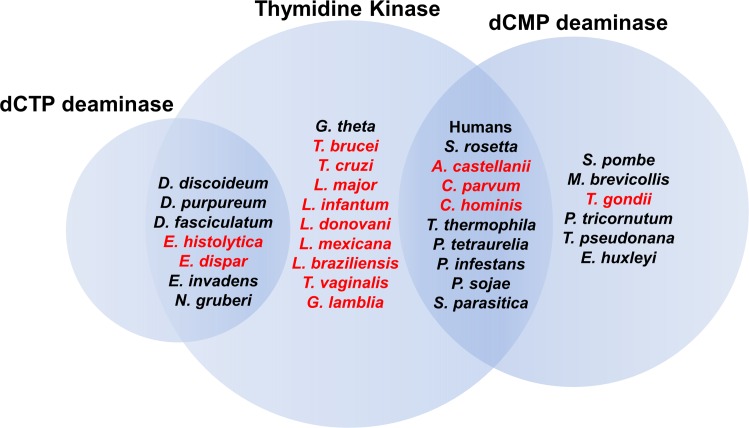
Venn diagram showing the distribution of TK, DCTD and dCTP deaminase in representative protists and higher eukaryotes. The overlapping regions represent organisms that possess both of the indicated genes.

To assess if TK essentiality was likely to extend to other pathogenic protozoa we utilized the KEGG pathway database to determine the distribution of TK and DCTD throughout eukaryotes (Fig 9). A striking disparity was observed within protists when compared to higher eukaryotes. The vast majority of higher eukaryotes possess both TK and DCTD, which may explain TK’s non-essential role in these organisms. In contrast, the kinetoplastids and a number of other protozoan human pathogens such as *Giardia* encode only TK, suggesting that TK may be essential in these organisms as well. We also note that several of the protists such as *Entamoeba histolytica*, which lack DCTD, instead encode dCTP deaminase, an enzyme found almost exclusively in bacteria. Similar to DCTD, the ability to deaminate dCTP to dUTP offers an alternative path from cytosine to thymine nucleotide pools and thus we would predict that TK would not be essential in these species. Interestingly, these organisms represent the only eukaryotic KEGG organisms that have dCTP deaminase.

## Discussion

*T*. *brucei* encodes a complete *de novo* pyrimidine biosynthetic pathway, as well as a number of pyrimidine salvage enzymes that were thought to be redundant based on the presence of the *de novo* pathway. Herein we describe the first comprehensive analysis of the role of the pyrimidine salvage enzymes in *T*. *brucei* and we show that while *T*. *brucei* is not auxotrophic for pyrimidines, both TK and CDA are essential for *in vitro* growth and TK is essential for infectivity *in vivo* as well. The finding that these enzymes are essential could not be explained by the current annotation of the pyrimidine pathway in *T*. *brucei*. Our mechanistic analysis of the *TK* and *CDA* null cell lines uncovered the existence of an interconversion network between the deoxypyrimidine nucleoside and nucleotide pools, including the presence of a previously unknown 5’-nucleotidase that converts deoxycytidine, deoxyuridine and thymidine nucleotides to their respective nucleosides. In the absence of TK or CDA to balance this 5’-nucleotidase activity, the metabolic cycle breaks down leading to dead-end buildup of deoxypyrimidine nucleosides and to cell death. The existence of this recycling pathway provides a mechanism for the parasite to interconvert and balance the relative levels of the deoxyuridine, deoxycytidine and thymidine pools whether they originate from the *de novo* pathway or through salvage. Our conclusions are supported by the following arguments.

Firstly, TK is essential for both *in vitro* growth and infectivity in a mouse model of *T*. *brucei* infection and for formation of dTTP despite the fact that *T*. *brucei* is not a pyrimidine auxotroph. Thus the essential role of TK is not to salvage externally acquired pyrimidine precursors. Our data clearly show that TK activity is required for its function and that it plays a key role in the synthesis of dUMP, even for cells grown in a pyrimidine-free environment. We found that the function of TK can be replaced by expression of human DCTD, which provides an alternative route to dUMP formation from dCMP in many higher eukaryotes [17, 18]. DCTD has been shown to be essential for cell cycle progression and formation of dTTP pools in eukaryotes that lack TK (e.g. *Schizosaccharomyces pombe*) [39]. These data suggest that DCTD and TK can have functionally redundant roles in contributing to dTTP pools, supporting our observation that TK is essential for formation of thymine nucleotides in *T*. *brucei*.

The next significant key to the puzzle came from analysis of metabolomic data from the *TK* c-null cell line. These data showed that even in the absence of external pyrimidines the TK substrates dUrd and dThd, as well as the dUrd precursor dCtd buildup, leading to a dead-end accumulation of these precursors away from the essential deoxynucleotide pools resulting in depletion of dTTP. In the absence of an exogenous supply of these nucleosides the current annotation of the *T*. *brucei* genome does not provide a mechanism for these nucleosides to be synthesized, suggesting the presence of a missing enzyme that catalyzes conversion of deoxynucleotides into deoxynucleosides. The findings that *CDA* null cells are auxotrophic for dThd or dUrd further support this hypothesis since based on redundancy in the pathway, CDA should not be essential under any conditions. Furthermore the *CDA* null data support the presence of an enzymatic link between the deoxycytidine-containing nucleotide pools and dCtd/dUrd, since either dUrd or Thd are required for growth of *CDA* null cells. These data are consistent with previous published untargeted metabolomics data showing that isotope-labeled glucose was incorporated into both dUrd and dThd, and thus that *T*. *brucei* was capable of synthesizing these nucleosides *de novo* [40]. Lastly, the inability of uracil to rescue the growth deficit of the *CDA* null cells shows that uridine phosphorylase is not able to efficiently convert uracil to dUrd, eliminating the only known potential source for dUrd biosynthesis in *T*. *brucei*. Uridine phosphorylase was previously suggested to be the source of dUrd, based on the isotope-labeled glucose study [40], but our result is instead consistent with previous reports that 5-fluorouracil and 5-fluoro-Urd are not substrates for this enzyme [10].

Thus taken together, our data lead to the conclusion that *T*. *brucei* encodes an unidentified 5'-nucleotidase that converts dCMP and dTMP to dCtd and dThd, respectively. We identified a number of potential candidate genes in *T*. *brucei* that could encode this activity, including a homolog of the *E*. *coli* HD protein YfbR and two strong candidates from the HAD-domain related family. Notably we showed that the *T*. *brucei* YfbR homolog encodes a metal dependent HD domain 5’-nucleotidase with broad substrate specificity functioning on all three pyrimidine deoxy-mononucleotides. Whether or not the *T*. *brucei* YfbR homolog is the only 5’-nucleotidase in *T*. *brucei*, or whether it is even the dominant enzyme with this capability remain open questions. Mammalian cells encode at least seven 5’-nucleotidases with overlapping specificities [41–43] and *E*. *coli* encodes minimally three, one each from the HD, HAD and SurE superfamilies [38]. Thus it is likely that other candidate *T*. *brucei* genes identified in our bioinformatics analysis will also display activity. In mammalian cells the 5’-nucleotidases have been shown to be required for regulation of cellular dNTP levels and to provide a mechanism to maintain balanced ratios between the pools, which is essential for high fidelity DNA synthesis [43]. Like the *T*. *brucei* HD-domain 5’-nucleotidase, all described nucleotidases from the various families exhibit broad substrate specificity. The broad specificity allows these enzymes to function in a ubiquitous capacity for interconversion of the nucleotide pools.

The finding of 5’-nucleotidase activity in *T*. *brucei* leads directly to the essentiality of both TK and CDA, as in their absence the dead-end buildup of pyrimidine nucleosides leads to depletion of pyrimidine deoxynucleotides and to cell death. Within this context, the ability of DCTD to rescue the *TK* null cell line suggests that DCTD is able to effectively compete with the 5'-nucleotidase for the dCMP pools, converting sufficient amounts to dUMP where it can be efficiently shunted to dTMP even in the absence of TK. The existence of the metabolic cycle involving TK, CDA and 5’-nucleotidase provides the cell with a mechanism to interconvert between the deoxyuridine, deoxycytidine and deoxythymidine pools allowing presumably for better regulation and balance of their relative levels. While dUMP can also be formed from UDP, this pathway is apparently not sufficient to keep up with dUMP needs in the face of the dead-end accumulation of the TK substrates in the absence of TK. However this pathway remains an important additional source of dUMP as null mutants of dUTPase have been reported to be thymidine auxotrophs [19].

Our metabolomic analysis also uncovered some additional insights into *T*. *brucei* metabolism and regulation. In the presence of an outside source of pyrimidines (non-dialyzed serum), the pyrimidine nucleosides dUrd, dCyd, dThd and thymine accumulated in the *TK* c-null cells to higher levels than for cells grown in pyrimidine-free medium (dialyzed serum). These data confirm that in the absence of TK there is dead-end accumulation of these nucleosides but they also suggest that uridine phosphorylase is not a significant drainage point for these pools. Thus *T*. *brucei* uridine phosphorylase primarily catalyzes conversion of Urd to uracil, while it is not capable of synthesizing dUrd (as described above), or using it efficiently as a substrate. This hypothesis is consistent with previous reports that the recombinant *T*. *brucei* enzyme is 10-fold more active on Urd than dUrd [20]. Our metabolomic data also suggest that one response of *T*. *brucei* to TK depletion is increased nucleoside transport despite the fact that the upregulated transport was unable to relieve the growth block. A similar accumulation in dUrd in the presence of normal serum was previously reported for *T*. *brucei* BSF treated with thymidylate synthase inhibitors [10] suggesting this is a common response to starvation of thymine nucleotides.

Finally, we noted that the levels of TCA intermediates were significantly perturbed in both the *TK* c-null and *CDA* null cells, including significant increases in α-ketoglutarate and homocysteine upon loss of TK expression or removal of thymidine from the *CDA* null cells. α-ketoglutarate is formed in the transamination reaction that generates L-Asp, which in turn is required for the first step in *de novo* pyrimidine biosynthesis, while homocysteine leads to formation of methionine then 5,10-methylene tetrahydrofolate, needed to convert dUMP to dTMP. Taken together with an observed decrease in carbamoyl phosphate, another precursor of the *de novo* pathway, the data suggest the cells may attempt to compensate for the loss of TK by increasing flux through the *de novo* pathway. Finally we also observed a significant decrease in acetyl-ornithine/acetyl-putrescine. It is not immediately apparent how these metabolites are synthesized, but their presence in *T*. *brucei* has been previously noted [40]. It is also not immediately clear what role they may play in pyrimidine biosynthesis, but both the synthesis and degradation of acetyl-ornithine can be catalyzed by aminotransferases, and in the case of its degradation this pathway links back to glutamate pools, and thus potentially to pyrimidine biosynthesis. The specific aminotransferases that catalyze these reactions are not annotated in the *T*. *brucei* genome, but aminotransferases have been reported to have broad and redundant substrate specificities in *E*. *coli* [44].

While a key aspect of our work was to elucidate the role of TK and CDA in linking the *de novo* pathway to synthesis of the deoxynucleotide pools, we have also validated TK as a drug target in *T*. *brucei* by showing that it is essential both *in vitro* and *in vivo*. The finding that the *TK* c-null cells cannot be rescued by exogenous pyrimidines shows it would not be possible for even an intracellular parasite to get around the block. Our work additionally showed that CDA is essential for *in vitro* growth of blood form *T*. *brucei*. While we did not determine if CDA is required for virulence *in vivo*, it remains a possibility provided that blood thymidine levels are below those required for rescue. Pyrimidine deoxynucleosides levels in human blood are reported to be ~10-fold below the EC_50_ that we measured for efficient rescue of *CDA* null growth. Furthermore, *T*. *brucei* has low affinity and/or poor efficiency transporters for deoxynucleosides [10], suggesting that *CDA* may be essential for infection in humans. However, additional studies will be needed to address this question conclusively.

The presence of multiple pathways to synthesize dUMP appears to be an important shared characteristic amongst many eukaryotic cells, with the data suggesting that some organisms require either TK or DCTD to link *de novo* biosynthesis to the thymine deoxynucleotides. Interestingly, our bioinformatics analysis shows that other single-celled eukaryotic pathogens, including all three disease-causing trypanosomatids, encode TK but lack DCTD. These data suggest that TK may be essential in these other pathogens and may potentially provide a path forward to develop drugs that have pan-activity against a range of human pathogens. However the essentiality in other organisms would be dependent on the presence of the 5’-nucleotidase activity and likely also on limited catabolism of dUrd back to uracil by uridine phosphorylase. In support, a homolog of the *T*. *brucei* HD domain 5’nucleotidase is present in *Leishmania* and *T*. *cruzi*. Furthermore *Leishmania major TK* null cell line showed severely reduced growth rates [45]. In contrast, deletion of TK from *Cryptosporidium parvum* was not lethal, which is predicted by the presence of both TK and DCTD [46]. The finding that human cells contain both TK and DCTD, and that TK is not essential in human cells [47] supports the potential for selectively targeting TK from the eukaryotic pathogens that lack DCTD. Thus in conclusion, the unexpected finding that TK is essential in *T*. *brucei* and its mechanistic role in supporting *de novo* pyrimidine biosynthesis has uncovered a unique opportunity for the potential development of a pan-trypanosomatid therapy.

## Materials and Methods

### Ethics statement

All animal care and experimental procedures were in accordance with the office of Laboratory Animal welfare (OLAW) guidelines provided by the National Institute of Health (NIH) USA (Assurance Number D16-00296) and the USDA (Registration Number 74-R-0072) as specified by the University of Texas Southwestern Medical Center Animal Care and Use Committee (IACUC) guidelines. Animal experiments were approved by the Ethical Review Committee at the University of Southwestern Medical Center and performed under the IACUC-2012-0021 protocol. All mice were housed in an animal facility with barrier SPF conditions.

### Gene accession numbers

*T*. *brucei* gene sequences were obtained from TriTrypDB and the gene accession numbers are as follows: TK (Tb927.10.880), CDA (Tb927.9.3000), HD-domain 5’-nucleotidase (Tb09.211.2190), TERT (Tb927.11.10190). The *Hs*TK1 (P04183) and *Hs*DCTD (P32321) amino acid sequences were obtained from UniProt, and *Hsv*TK was derived from Addgene plasmid #48356.

### *T*. *brucei in vitro* growth

Experiments were performed using *T*. *brucei* BSF SM cells genetically manipulated to express T7 RNA polymerase and the Tet repressor (TetR) [29]. Cells were grown in HMI-19 medium supplemented with 10% fetal bovine serum (FBS) at 37°C in 5% CO_2_. HMI-19 is a modified medium that we previously reported [9]. It was designed to contain more physiologically relevant purine and pyrimidine levels and it is supplemented with only 10 μM hypoxanthine and no added thymidine, except that present in FBS. To obtain completely pyrimidine-free conditions, normal FBS was replaced with dialyzed FBS in media where indicated. All cells were maintained in exponential growth (10^5^−10^6^ cells/mL). SM cells were maintained in G418 (2.5 μg/mL) to retain the T7 polymerase and TetR. *TK* and *CDA* RNAi and knockout lines were cultured in the appropriate antibiotic depending on the transfected plasmid at the following concentrations unless otherwise stated: 2.5 μg/mL G418 (Life Technologies), 2.5 μg/mL blasticidin (InvivoGen), 2.5 μg/mL phleomycin (InvivoGen), 1–2 μg/mL hygromycin (Sigma), 0.1 μg/mL puromycin (Sigma), and 1 μg/mL Tet (RPI). For all nucleoside supplementation experiments 100 mM stocks of sterile filtered deoxyuridine (Sigma), thymidine (Sigma), uridine (Sigma), and uracil (Sigma) were added to cultures at concentrations indicated. All c-null lines were supplemented with 1 μg/mL Tet daily to maintain steady expression of Tet-regulated proteins. For pyrimidine and Tet free conditions, cells were washed (3 x 20 mL) with the appropriate media prior to beginning the growth experiments. For evaluation of growth rates, cells were washed and replated in media containing no antibiotics at a density of 20,000 cells/mL and diluted over the course of the study to maintain exponential growth. Cell density was determined using a hemocytometer (Bright-Line) with a lower limit of detection of 10^4^cells/ml. Two technical replicates were averaged for each counted sample. Total cell numbers were calculated by multiplying cell density by the dilution factor and volume [48].

### Transfection of *T*. *brucei*

For each transfection, parasites (10^7^) were suspended in Human T Cell Nucleofector Buffer (Lonzo)(100 μL) containing NotI linearized vector (5 μg) or purified PCR product (1 μg) as described [49]. All transfected DNA was confirmed by sequencing prior to transfection. Negative controls cells transfected with buffer only were prepared alongside samples to optimize selection conditions. Cells were transfected using protocol X-001 on the Amaxa Nucleofector (Lonza) and then transferred to media (25 mL) and allowed to recover 8 h prior to addition of selection antibiotics. Two dilutions (1:20 and 1:40) of culture, containing selection antibiotics, were plated in 24-well plates at 2 mL/well. Negative control plates were monitored throughout the experiment to ensure selection was achieved. After several days, wells containing a cell density of about 10^6^ cells/ml were selected for generation of clonal lines by limiting dilutions.

### Generation of *T*. *brucei* TK conditional null cell lines

The *T*. *brucei* TK and human (*Hs*) DCTD expressing *TK* c-null cell lines were generated utilizing the fusion PCR method [49, 50]. Cloning primers are shown in S2 Table. The first *TK* allele was replaced by the *HYG* resistance gene by PCR fusion of *TK* 5’ and 3’ UTRs to *HYG*. The *HYG* resistance gene was derived from the pLew90 vector [29] (a gift from George A.M. Cross). UTRs were amplified from genomic DNA isolated from SM cells. To generate the *TK* single allele knockout (SKO) line the purified PCR product was transfected into SM cells and hygromycin resistant cells were selected in medium containing G418 and hygromycin. The *TK* SKO line was then used to generate the remaining cell lines. A Tet-regulated vector containing either FLAG-tagged *Tb*TK or FLAG-tagged *Hs*DCTD was cloned as follows. The *T*. *brucei TK* or the human *DCTD* genes were PCR amplified from *T*. *brucei* SM genomic DNA or from human cDNA synthesized from RNA extracted from a human breast adenocarcinoma cell line (MDA-MB-231), respectively. For both constructs, the forward direction PCR primer contained a flanking 5’ HindIII restriction site and an N-terminal FLAG tag; the reverse direction primer contained a flanking 3’ BamHI restriction site. The restriction digested PCR products were ligated into the pLew100v5-phleo vector (a gift from George A.M. Cross). The pLew100v5-phleo vector was linearized by the NotI restriction enzyme to facilitate integration into the rRNA spacer region. Linearized vector (5 μg) was transfected into the *TK* SKO cell line and selected for resistance to phleomycin. The resulting clones were screened to identify those with the tightest level of Tet regulation of the ectopically expressed protein. Finally, the remaining *TK* allele was replaced by a *PAC* resistance gene synthesized by GenScript. The *PAC TK* UTR fusion product was generated as described above and transfected into *TK* SKO cells expressing either *T*. *brucei* TK or *Hs*DCTD grown in Tet containing medium for 2 days prior to transfection. *TK* c-null cells were selected and maintained in G418, phleomycin, hygromycin, puromycin, and Tet (added daily). PCR primers flanking the 5’ and 3’UTRs were used to confirm that the *TK* gene had been replaced by the selectable markers.

### Generation of the TK RNAi *T*. *brucei* BSF cell line

RNAit (http://trypanofan.bioc.cam.ac.uk/software/RNAit.html) was used to identify a suitable 566 bp region located in the *TK* 3’UTR. The 3’UTR was targeted to allow compatibility with *Tb*TK rescue plasmids (described below), that utilize instead the *ALD* 3’UTR. Genomic DNA isolated from SM cells was used as template for PCR amplification of the target region and TA cloned into the Gateway vector pCR8/GW/TOPO (Life Technologies). The Tet inducible stem loop was created by addition of Gateway LR Clonase to a reaction containing both the Gateway vector (100 ng) and pTrypRNAiGate vector (100 ng) [51]. *TK* SKO (*hyg*) cells were transfected with the vector and integration into the rRNA spacer region was selected using phleomycin. For studies of the effects of *TK* knockdown, Tet was added daily to induce formation of the hairpin leading to knockdown of *TK* mRNA. Cells were grown in the absence of other antibiotics for these studies.

### Cloning of RNAi rescue constructs and TK mutants

The *Tb*TK rescue construct under control of the Tet promoter was generated using the same approach described above for the c-null cell line except that an N-terminal AU1-tag was included instead of a FLAG-tag to allow detection of the expressed protein. The gene encoding the *Hs*TK open reading frame was synthesized by GenScript and cloned into the pUC57 vector, which was used for subsequent PCR amplification to generate the FLAG-tagged *Hs*TK rescue construct. *Hsv*TK was amplified from the pHJ17 Hyg-TK-loxP vector (Addgene). To generate the *Tb*TK E286A and *Hs*TK K32I mutants, both wild-type genes were subcloned into the pCR2.1-TOPO TA vector (Invitrogen). Complimentary PCR primers containing the desired point mutation were synthesized. Phusion polymerase (NEB) was used to amplify the entire vector according the to the following parameters: initial denaturation at 95°C for 30 s followed by 18 cycles of denaturation at 95°C for 30 s, annealing at 68°C for 1 min, and amplification at 72°C for 5 min. Each reaction (50 μL) was treated with DpnI (NEB)(1 μL) overnight at 37°C followed by transformation into T10 cells and selection with ampicillin (100 μg/ml). Clones were sequenced using M13 primers. All constructs contained flanking 5’ HindIII and 3’ BamHI restriction sites that permitted ligation into the pLew100v5-bsd vector[49]. The vectors were linearized with NotI and transfected into *TK* RNAi cells as described above.

### Generation of the *T*. *brucei* CDA null cell line

SM cells were transfected with the *HYG* resistance gene flanked by the *CDA* 5’ and 3’ UTRs, generated by fusion PCR as described above, to generate the SKO in medium containing G418 and hygromycin. For the remaining allele, a fusion PCR product containing the *PAC* resistance gene was transfected into the SKO cells. Null cells were selected in growth medium containing G418, hygromycin, puromycin, and Thd (500 μM). PCR primers flanking the *CDA* 5’ and 3’ UTRs were used to confirm replacement of the *CDA* alleles.

### Liquid chromatography-tandem mass spectrometry (LC-MS/MS) metabolomics analysis

*T*. *brucei* TK expressing *TK* c-null cells were grown with or without Tet for 24 h and *CDA* null cells were grown with or without thymidine (0.5 mM) for 12 h. Cells (10^8^) were harvested by centrifugation (3500 RPM, 5 min) and then washed in cold PBS (50 mL). Washed pellet was resuspended in 1 mL pre-chilled (-80°C) 80% methanol and incubated on ice for 10 min. The cell extract was centrifuged (16,000 x g, 4°C, 20 min) to remove insoluble debris and 0.9 mL of supernatant was dried using a vacuum centrifuge. Samples were stored at -80°C prior to analysis. For pyrimidine-free studies, a starter culture was washed, as described above, and grown in pyrimidine-free medium for 48 h prior to the start of the experiment. Targeted metabolite profiling by LC-MS/MS was performed as previously described allowing for detection of ~ 130 standard metabolites [52]. While this method allowed for quantitation of many key nucleosides and bases, the deoxynucleotides were not profiled as they are not part of the trained set of the facility. In order to attempt to identify these metabolites we isolated a larger cell number (5×10^8^cells) and again used targeted LC-MS/MS for detection as described [53]. Levels of deoxynucleotides in wild-type control SM cells were barely detectable so null lines were not analyzed.

### Thymidine triphosphate (dTTP) quantitation by enzymatic assay

Because we were unable to quantitate dTTP by LC-MS/MS approaches we employed a previously reported enzymatic assay that monitors Klenow DNA polymerase catalyzed incorporation of ^3^H-dATP into synthetic oligonucleotides in a reaction that is proportional to the amount of dNTP [54, 55]. Through use of a standard curve the targeted dNTP concentration in the sample was determined. The oligo template that was used for the assay was as reported [54]. To prepare cell extracts, *TK* c-null cells were grown with or without Tet for 24 or 48 h in standard HMI-19 media supplemented with normal FBS and were harvested by centrifugation (3500 RPM, 5 min) and washed once with PBS. The pellets were resuspended in 60% methanol (250 μL) and incubated at -20°C overnight. Cell extracts were placed in a boiling water bath for 5 min, centrifuged (16,000xg, 20 min, 4°C), and then the soluble fraction was dried by vacuum centrifugation. Dried extracts were dissolved in 100 μL sample buffer (40 mM Tris-HCl pH 7.4, 10 mM MgCl_2_). Each reaction (100 μL) contained 40 mM Tris-HCl (pH 7.4), 10 mM MgCl_2_, 5 mM DTT, 0.25 μM oligonucleotide template, 1.5 μg RNase A, 0.25 μM ^3^H-dATP (ARC- 17.2 Ci/mmol), 0.3 units Klenow Fragment (NEB), and cell extract (10 μL) or dTTP standard. Reactions were incubated at 37°C for 1 hr before spotting (85 μL of reaction) onto DE81 paper disks (23 mm-GE Healthcare), which were then air dried. Disks were washed (3 x 10 min) with 25 mL 5% Na_2_HPO_4_, rinsed once with water (25 mL) and absolute ethanol (15 mL). Dried disks were placed in scintillation liquid and radioactivity was measured by scintillation counting. A standard curve was generated with 0–4 pmol dTTP (New England BioLabs).

### Nucleotide sugar analysis

*T*. *brucei* cells (2x10^7^) were pelleted by centrifugation (3500 RPM, 5 min), washed in cold PBS once and then resuspended in 1 mL pre-chilled methanol. Samples were freeze-thawed in liquid nitrogen, placed on ice for 10 min, and centrifuged (16,000 x g for 10 min at 4°C) to remove insoluble debris. A portion of the supernatant (0.8 mL) was vacuum dried and the residue resuspended in 40 mM sodium phosphate, pH 7.4. Samples were syringe filtered (Millex GV 0.22 μM–Millipore) to remove fine particles. High-Performance Anion Exchange Chromatography (HPAEC) analysis was performed as published [56] but with a modified elution gradient that was optimized for the separation of UDP-GalNAc, UDP-GlcNAc, UDP-Gal and UDP-Glc. Briefly, chromatography was performed on a Dionex ICS3000 HPAEC system with a CarboPac PA1 analytical column (4 mm Å~ 250 mm) and guard column (4 mm Å~ 50 mm). The method was performed with eluents 1 mM NaOH (E1) and 1 M NaOAc, 1 mM NaOH (E2) as follows: 0 min—20% E2, 10 min—45% E2, 25 min—45% E2, 35 min—45% E2, 40 min—100% E2, 50 min—100% E2, 55 min—20% E2, 65 min—20% E2. Equal amounts of sample (approximately 50 Mio cells) were analyzed within each experiment and the signal divided by cell count. Synthetic standards were acquired from Promega.

### RNA and DNA purification

DNAzol (Molecular Research Center) was used to isolate genomic DNA from *T*. *brucei* cells. Typically, 5x10^7^ cells were collected for DNA extraction using guidelines recommended by the manufacturer. Total RNA was extracted from samples (3x10^7^cells) using TRIzol (Invitrogen), following the manufacturer’s protocol.

### Quantitation of mRNA by qPCR

As described above, total RNA was isolated from samples and treated with DNaseI (Invitrogen) to eliminate gDNA contamination. A cDNA reverse transcription kit (Applied Biosystems) was used to synthesize cDNA for downstream analysis. Relative mRNA abundance was quantified using iTaq SYBR Green Supermix with ROX (Bio-Rad) utilizing a standard curve for each set of primers per experiment. For all experiments, *TERT* was used as a reference gene [57]. Data was collected on the CFX96 (Bio-Rad) and analysis was performed using the Pfaffl method [58].

### Virulence studies in mice

Mice (C57BL/6J) were purchased from Wakeland Laboratory (UT Southwestern) and were group-housed in filter-top cages. The animal facility has standard laboratory conditions: 21 to 22°C ambient temperature and a 12 h light/12 h dark cycle. Chow and water were available *ad libitum*. Both doxycycline (Dox) water and water only (controls), were supplemented with 0.1% saccharin to ensure animals drank the Dox supplemented water. Mice from each group were introduced to the study drinking water 2 days prior to infection. Water bottles were protected from light and replaced every 2–3 days. Mice drank approximately 12.5 mL of water daily. Mice (8 weeks old, n = 6) (12 in total, 3 per study arm) were infected intraperitoneally with 10^3^ SM or *TK* c-null parasites ± Dox. Prior to the infection *TK* c-null parasites were propagated in +Tet conditions to ensure parasite viability at the start of the study. Mice were monitored for parasitemia starting three days post-infection by collecting 1 μL of blood from the tail in a 1:150 dilution of medium and counted using a hemocytometer as described [59]. Mice were monitored for 30 days post infection.

### Western blot analysis

Cells (5x10^7^) were harvested by centrifugation (3500 RPM, 5 min), washed twice in cold PBS (10 mM Na_2_HPO_4_, 1.8 mM KH_2_PO_4_, 137 mM NaCl, 2.7 mM KCl, pH 7.4), and resuspended (50 uL) in trypanosome lysis buffer (50 mM Hepes, pH 8.0, 100 mM NaCl, 5 mM, β-mercaptoethanol, 2 mM PMSF, 1 mg/mL leupeptin, 2 mg/mL antipain, 10 mg/mL benzamidine, 1mg/mL pepstatin, 1 mg/mL chymostatin). Cells were lysed by 3 freeze/thaw cycles. Insoluble debris was pelleted by centrifugation (16,000 x g, 20 min, 4°C) and the soluble fraction was collected. The Bio-Rad Protein Assay reagent was used to determine protein concentration with bovine serum albumin (BSA) used to generate a standard curve. Total protein (20 μg) was resolved by 12% SDS-PAGE and transferred to a PVDF membrane using the Mini Trans-Blot Cell (BioRad). Membranes were blocked by 5% non-fat dry milk in Tris-buffered saline (TBS) (20 mM Tris (pH 7.6), 150 mM NaCl) for 1 h then incubated with a primary antibody in 5% milk and TBS-T (TBS + 0.1% Tween-20) overnight at 4°C. The following dilutions were used for each primary antibody: αFLAG 1:1000 (Rabbit polyclonal-Thermo Fisher), αAU1 1:1000 (Mouse monoclonal – Covance), α*Hs*TK 1:500 (Mouse monoclonal—Thermo Fisher), α*Tb*BiP 1:100,000 (Covance). For detection of the primary antibody, the membrane was incubated in a 1:10,000 dilution of a Protein A-HRP conjugate (Abcam) in TBS-T (5% milk) for 1 hour at room temperature. The membrane was washed 5 x 5min with TBS-T and incubated in SuperSignal West Pico Chemiluminescent Substrate (Thermo Scientific) for 5 minutes. The membrane was visualized by the ImageQuant LAS 4000 (GE).

### Data analysis

Graphs were generated in GraphPad Prism version 7.0a for Mac, GraphPad Software, San Diego California USA (www.graphpad.com), and statistical analysis was performed as indicated in the figure legends.

### Genomic analysis to identify possible nucleotidyltransferase enzymes

The KEGG (Kyoto Encyclopedia of Genes and Genomes[60, 61]) pyrimidine metabolic pathway highlights two enzymatic reactions (EC 3.1.3.5 and EC 3.1.3.89) that perform the 5'-(deoxy)nucleotidase activity required to produce pyrimidine deoxynucleosides in other organisms. We searched all reviewed UniProtKB [62] entries with these two described enzyme activities, identifying 561 genes with EC 3.1.3.5 activity and 70 genes with EC 3.1.3.89 activity. We sorted the identified genes according to their assigned PFAM domains [63], keeping nine representative sequences from each unique PFAM, which correspond to five different fold groups (Table 1). For stringent identification of *T*. *brucei* protein sequences with potential 5' nucleotidase activity, the representative sequences were used as queries to search the NCBI NR database using PSI-BLAST [64] (5 iterations, E-value cutoff 0.001), storing the resulting position-specific scoring matrix as a checkpoint file for re-initiating BLAST against a database of protein sequences from the *T*. *brucei* genome (E-value cutoff 1). Identified *T*. *brucei* protein sequences were assigned PFAM domains using batch CD search [65], keeping those sequences with PFAM domains described as possessing 5’-nucleotidase activity. To identify all potential *T*. *brucei* protein sequences with domains related to those described as having 5'-nucleotidase activity, we queried the *T*. *brucei* genome using RPS-BLAST (E-value cutoff 0.006) with a library of sequence profiles downloaded from the conserved domain database (CDD ID in S1 Table) corresponding to each described 5'-nucleotidase PFAM in Table 1. Identified sequences were crosschecked for the presence of the query domain using batch CD search [65] or HHPRED [66, 67], reporting the positive hits using the initial RPS-BLAST E-values in S1 Table. Additional methods details are provided in S1 Text.

### Cloning, expression and enzymatic assay of *T*. *brucei* 5’-nucleotidase (HD-fold, YfbR-like)

The DNA sequence for the *T*. *brucei 5’-nucleotidase* ***(***HD-fold, YfbR-like) was obtained from TriTryDB (Tb09.211.2190). The *E*. *coli* codon optimized gene was synthesized by GenScript. PCR was used to generate flanking BsaI and XbaI restriction sites that allowed for cloning into the pE-SUMO(KAN) vector (LifeSensors, Malvern, PA) and expression as a N-terminal His_6_-SUMO fusion protein. The nucleotidase pE-SUMO vector was transformed into BL21 cells. Cells were cultured in 2L LB-KAN (50 μg/mL) media at 37°C until OD_600_ 0.7, then cooled to 16°C and induced by 500 μM IPTG (Isopropyl β-D-1-thiogalactopyranoside) for 16 h. Cells were collected by centrifugation and suspended in buffer A (500 mM NaCl, 50 mM HEPES pH 7.5, 5 mM imidazole, 5% glycerol, 5 mM 2-mercaptoethanol) supplemented with 2 mM PMSF (phenylmethane sulfonyl fluoride) and a protease inhibitor cocktail (1 mg/mL leupeptin, 2 mg/mL antipain, 10 mg/mL benzamidine, 1 mg/mL pepstatin, 1 mg/mL chymostatin). Cells were lysed by cell disruptor and the cell debris removed by centrifugation. Supernatant was applied to a HisTrap HP column (GE Healthcare) and washed with buffer A. The His_6_-SUMO nucleotidase fusion was eluted using a gradient of 5–45% buffer B (500 mM NaCl, 50 mM HEPES pH 7.5, 5% glycerol, 5 mM 2-mercaptoethanol, 500 mM imidazole). Fractions were analyzed by SDS-PAGE and those containing the *T*. *brucei* HD domain 5’-nucelotidase were pooled, concentrated (10 kDa MWCO Millipore) and dialyzed against buffer A. The His_6_-Sumo tag was removed by overnight incubation at 4°C with His_6_-tagged Ubiquitin-like-specific protease 1 (ULP1) as described [68]. After incubation the mixture was applied to a second HisTrap HP column and cleaved 5’-nucelotidase was collected in the flow-through, while His_6_-tagged ULP-1 and impurities were retained on the resin. The flow through was concentrated and protein purity was assessed to be >98% by SDS-PAGE analysis (S14 Fig). This tagless protein was used to collect the data shown in Fig 8. To provide an alternative purification method, His_6_-Sumo tagged 5’-nucleotidase eluted from the first Ni^+2^ column was further purified by Gel Filtration chromatography on a SuperDex 200 Prep Grade column (GE Healthcare) using buffer A. The His_6_-Sumo tagged 5’-nucleotidase showed similar activity (within 2-fold) of the untagged version. Protein concentrations were determined by Bradford Assay (Bio-Rad).

*T*. *brucei* 5’-nucleotidase activity was assessed using an endpoint assay for released inorganic phosphate (P_i_) using Malachite Green as the detection reagent as described [38, 69]. Briefly, each reaction (160 μL) contained 50 mM HEPES pH 7.5, 0.5 mM CoCl_2_, 1 mM substrate, and enzyme. The reactions were incubated for 10–30 min (to confirm linearity) at 37°C and then 50 uL was treated with 100 mM EDTA. Malachite green reagent (150 μL) was added to each quenched reaction and incubated at room temperature for 5 min prior to measurement at 650 nm. A range of substrate (0.1–1.0 mM), metal (0.5–10 mM) and enzyme (25–100 nM) concentrations were tested to confirm linear dependence on enzyme concentration and to confirm that the reaction rate versus substrate curve was a saturable process. The production of P_i_ was measured at 650 nm. Absorbance was converted to μmoles of Pi using a standard curve generated using phosphate standard (Cayman Chemical) ranging from 0–100 μM. For the substrate and metal ion specificity studies shown in Fig 8, assays were run with 100 nM enzyme for 20 min using a substrate concentration of 1 mM. Metal ion concentrations are indicated on the graph. All data were collected in triplicate.

## Supporting Information

S1 Appendix (XLSX)

S1 Text Supplemental Methods. (PDF)

S1 Table *T*. *brucei* sequences with identified 5'-nucleotidase signatures. (PDF)

S2 Table PCR and cloning primers. List of cloning primers and corresponding sequences (5’-3’) used throughout the study. The primer names contain information regarding their target, restriction sites, and functions.
(PDF)

S1 Fig PCR analysis confirms replacement of *TK* alleles with selectable markers. Amplification of the *TK* locus using primers flanking the *TK* 5’ and 3’ UTRs (A,B), selectable markers(A,B), and *TK* ORF (B). The *TK* locus was amplified from genomic DNA extracted from *TK* c-null and WT SM cells and the selectable markers were amplified from *TK* c-null genomic DNA. The *TK* gene was amplified from both the WT SM and human DCTD expressing *TK* null genomic DNA.
(TIFF)

S2 Fig Infection of mice with *TK* c-null cells and wild-type SM cells. Graphs show parasitemia levels (cells/ml) in blood collected the first 6 days post infection for three mice per each group. A. SM cells ±Dox. B. *TK* c-null cells ±Dox. Mouse #2 in the TK c-null +Dox arm was delayed in the course of infection and did not succumb to parasitemia until day 18. Parasites were not observed at any time over the 30 days in the TK c-null -Dox treated animals.
(TIFF)

S3 Fig Growth analysis of *TK* RNAi cells (±Tet) expressing Tet regulated *Hsv*TK. **A** *TK* RNAi cells (±Tet) expressing Tet regulated *Hsv*TK grown in parallel with the *TK* RNAi control (±Tet). B. Growth analysis of TK RNAi cells (±Tet) expressing Tet regulated *Tb*TK or *Hsv*TK grown in the presence or absence of ganciclovir (GCV-50 μg/ml). C. qPCR analysis of relative TK expression in *Hsv*TK rescue cells 48 h after Tet supplementation compared to SM (WT) cells. *Tb*TK expression is normalized with TERT expression and error bars represent standard error of the mean (SEM) calculated from triplicate data. All growth experiments were performed in HMI-19 media and the error bars represent the standard deviation (SD) for biological triplicates.
(TIFF)

S4 Fig Amino acid sequence alignment (Clustal Omega version 1.2.2) of TKs from select species. *E*. *coli* (*Ec*-P23331), Humans (*Hs – P04183*), *Mus muscaris* (*Mm – P04184*), *Leishmania donovani* (*Ld – Q4QC75*), inactive N-terminal *Tb*TK domain, and active C-terminal *Tb*TK domain. Residues highlighted in green represent highly conserved residues in active TKs selected for the mutagenesis studies (*Tb*TK E286A and *Hs*TK K32I).
(PDF)

S5 Fig Treatment of *TK* RNAi cells grown minus Tet and various nucleoside supplements. Control studies for data reported in Fig 4 to demonstrate that exogenous nucleosides do not affect growth of cells that are expressing TK. A. Cells were grown in normal serum based media plus 0, 1 or 5 mM dUrd in the absence of Tet. B. Cells were grown plus or minus 5 mM dUrd or Urd. C. Cells were grown plus 0, 0.12, 0.5 or 1.0 mM dThd. Error bars represent the standard deviation (SD) for biological triplicates.
(TIFF)

S6 Fig Uracil supplementation is unable to rescue loss of TK in *TK* c-null cells. A. Growth analysis of *TK* c-null cells (±Tet) in HMI-19 media supplemented with uracil (μM). Error bars represent the standard deviation (SD) for biological triplicates.
(TIFF)

S7 Fig Metabolomic profiling of *TK* c-null cells. A-B. Heat maps represent average fold changes (-Tet 24hr/+Tet) in relative metabolite abundance. A) normal serum (NS); B) dialyzed serum (DS). For NS, pink indicates fold changes exceeding the scale (fold change > 10).
(TIFF)

S8 Fig Metabolomic profiling of *TK* c-null cells: changes in TCA cycle and polyamine metabolites. Fold change (-Tet 24h/+Tet) for A, B) TCA cycle metabolites NS vs DS or for C, D) polyamines NS vs DS. Error bars represent SEM calculated from biological triplicate data. Metabolites that showed a significant difference between the conditions are marked * P<0.05, ** P<0.01. Statistical significance was calculated as described in Fig 6 of the main paper.
(TIFF)

S9 Fig Metabolomic profiling of *TK* c-null cells: changes in amino acid metabolism. A-B. Fold change (-Tet 24h/+Tet) in relative abundance of amino acids and related metabolites grown in NS and DS. Error bars represent SEM calculated from biological triplicate data and statistical analysis is as described in Fig 6 and S7 Fig.
(TIFF)

S10 Fig PCR analysis confirms replacement of *CDA* alleles with selectable markers. A. Amplification of the *CDA* locus using primers flanking the *CDA* 5’ and 3’ UTRs, selectable markers, and *CDA* ORF. The *CDA* locus was amplified from genomic DNA extracted from *CDA* null and SM cells. The selectable markers were amplified from *CDA* null genomic DNA. The *CDA* gene was amplified from both SM and *CDA* null genomic DNA. B. The puromycin selection marker and *CDA* gene possessed similar sized PCR products. The PCR product corresponding to the amplified puromycin resistance gene contained a unique EcoRV restriction site, allowing for discrimination between the two PCR products.
(TIFF)

S11 Fig Bar graph representation of metabolomic profiling of *CDA* null cells. Fold change in relative metabolite abundance comparing CDA null cells (-Thd 12h/+Thd) grown in media containing dialyzed FBS in the absence of Thd for 12 h versus cells grown in media supplemented with 0.5 mM Thd. Panels represent A) nucleotides B) polyamines C) amino acids and D) TCA cycle intermediates. Error bars represent SEM calculated from biological triplicate data. Metabolites that showed a significant difference between the conditions are marked * P<0.05, ** P<0.01. Statistical significance was calculated as described in Fig 6.
(TIFF)

S12 Fig Heat map representation of metabolomic profiling of *CDA* null cells. Heat map represent average fold changes (-Thd 12hr/+Thd) in relative metabolite abundance. Cells colored gray represent metabolites with fold decreases greater than 10. The presence of thymine in the +Thd treated cells is likely caused by contamination of the commercial Thd source with thymine.
(TIFF)

S13 Fig Sequence alignment of *T*. *brucei* HD-domain 5’-nucleotidase, with representative eukaryotic and bacterial homologs. Sequences closest to the protein encoded by *T*. *brucei* Tb09.211.2190 were collected by BLAST against the RefSeq database, with HDDC2 being the closest representative in human. We generated a multiple sequence alignment with PROMALS-3D using the structures of human HDDC2 (4dmb), *E*.*coli* YfbR (2pau), *M*. *magnetotacticum* (3kh1), *P*. *furiosus* (1xx7), and *A*. *fulgidus* (1ynb); together with select HDDC2 homologs defined by HomoloGene and representative protists close to *T*.*brucei*. The HDDC2/YfbR sequences are ubiquitous, with representatives all three domains of life. Representatives are labeled to the left by PDB ID or NCBI accession, followed by species, and colored according to taxonomy: bacteria (blue labels), archaea (red labels), and eukaryota (animals black, fungi orange, plants green, and protists magenta). Secondary structures are indicated above the alignment, with H representing helix. Active site residues defined in YfbR (2pau) are invariant and are labeled above the alignment, including metal coordinating residues (H), nucleotide phosphate binding (P), nucleotide ribose binding, (R), and catalytic (C). Residue positions are highlighted according to conservation: including mainly hydrophobic (yellow) and small (gray) positions that dictate structure, and mainly polar (black) positions that dictate function. The YfbR structure (2pau) was of the E72A mutant enzyme so the alignment shows the residue as an Ala (red) even though the wild-type enzyme contains a Glu at this position.
(PDF)

S14 Fig SDS-PAGE analysis of purified *T*. *brucei* HD-domain 5’-nucleotidase. The gel shows the final purified 5’-nucleotidase (lane 2) after cleavage and removal of the His_6_-Sumo tag. 10 μg of 5’-nucleotidase was loaded on the gel. The molecular weight of protein standards in lane 1 are shown.
(TIF)
