# Supplementary material for: Pyrimidine Salvage Enzymes Are Essential for De Novo Biosynthesis of Deoxypyrimidine Nucleotides in Trypanosoma brucei
Source: PLoS Pathog. 2016 Nov 7;12(11):e1006010. doi: 10.1371/journal.ppat.1006010 (PMC5098729; doi:10.1371/journal.ppat.1006010)
Supplement: S1 Text — (PDF) [file ppat.1006010.s002.pdf]

Supplemental Methods

Identifying nucleotidyltransferase enzymes in the T. brucei genome

This section provides additional description of the analysis of each of the families identified in Table 1 that are present in *T. brucei*, and which led to identification of the top candidates for likely 5’-nucleotidyltransferase activity in *T. brucei*. The full list of potential genes is given in Table S1.

Phosphoglycerate mutase-like superfamily. The phosphoglycerate mutase-like superfamily includes a branch of histidine acid phosphatases (PF00328: His_phos_2) that share a conserved catalytic core containing a histidine residue that becomes transiently phosphorylated during the reaction[1]. Diverse activities associated with this histidine phosphatase branch 2 include phytase, glucose-1-phosphatase, and multiple inositol polyphosphate phosphatases, in addition to the ecto 5' nucleotidase activity (converting extracellular AMP to adenosine) described for the human ACPP gene product [2]. We identified nine *T. brucei* gene products containing histidine phosphatase superfamily signatures (Table S2). Several of these sequences are annotated as membrane-bound acid phosphatases (Tb11.01.4701, Tb11.01.3610, and Tb10.6k15.3560) or acid phosphatase (Tb10.6k15.3720) and contain predicted signal peptides, suggesting that they might function in a similar extracellular capacity as human ACPP. The remaining proteins annotated as glycerolphosphate mutase, phosphoglycerate mutase-like, fructose-6-phosphate2-kinase, or two hypothetical proteins are more closely related to the other branch of the phosphoglycerate mutase-like superfamily.

Metallo-dependent phosphatases family. The metallo-dependent phosphatases (MPPs) represent another large and diverse superfamily of enzymes with a conserved catalytic domain containing an active site coordinating two metal ions. Activities assigned to the superfamily include phosphoprotein phosphatases, Mre11/SbcD-like exonucleases, Dbr1-like RNA lariat debranching enzymes, YfcE-like phosphodiesterases, purple acid phosphatases, YbbF-like UDP-2,3-diacylglucosamine hydrolases, and acid sphingomyelinases. The human 5' nucleotidase representative, NT5E or CD73, converts extracellular AMP to adenosine terminating the action of nucleotide signals in the nervous system[3]. The NT5E sequence includes an N-terminal MPP superfamily catalytic domain and a 5'-nucleotidase C-terminal domain that mediates dimerization and binds nucleotide-derived compounds. Among the 33 identified *T. brucei* MPP sequences, none contain the 5'nucleotidase C-terminal domain nor are they identified by the specific CD73 sequence signature. The MPP sequences include 21 annotated as various serine/threonine protein phosphatases, 3 annotated as protein phosphatases, one diadenosine tetraphosphatase, one DNA repair protein, one vacuolar sorting protein, and 6 hypothetical proteins. Among the hypothetical MPP-containing proteins, two contain specific phosphoprotein phosphatase signatures, one contains a specific acid sphingomyelinase signature, one contains a specific lariat debranching enzyme signature, and the remaining two are distantly related to the MPP catalytic domain.

Haloacid dehalogenase family. The haloacid dehalogenases (HAD) represent a vast superfamily that includes both carbon and phosphorus hydrolases. Described activities include 2-haloalkonoate dehalogenase, epoxide hydrolase, phosphoserine phosphatase, phosphomannomutase, phosphoglycolate

phosphatase, and P-type ATPase, among others. Experimentally characterized activities of over twenty soluble HADs encoded in the *E. coli* genome [4] displayed broad and overlapping substrate specificities towards carbohydrates, nucleotides, organic acids, coenzymes, and small phosphodonors. Similar sets of *E.coli* HADs tended to act upon sets of structurally related substrates, with a group of three nucleotidases, YrfG, YjjG, and YieH, preferentially hydrolyzing purine nucleotides (GMP and IMP), pyrimidines (UMP, dUMP, and dTMP), and both purines and pyrimidines, respectively. Nucleotidase activity of a fourth *E. coli* protein, NagD, displayed unusually broad substrate specificity, functioning on deoxyribo- and ribonucleoside tri-, di-, and monophosphates, as well as polyphosphate and glucose-1-P. Among the 16 HAD domain-related sequences identified in the *T. brucei* genome, 11 represent hypothetical proteins without annotated functions. Only a single one of the hypothetical sequences (Tb09.211.1880) retains a specifically defined nucleotidase signature (corresponding to Pfam13344: Hydrolase_6) that is also closely related to the broad specificity *E. coli* NagD, while three others are only distantly related to HAD domains (Tb10.389.1370, Tb11.01.4360, and Tb11.v4.0008).

HD domain family. The HD domain superfamily represents metal dependent phosphohydrolases with a conserved 'HD' motif that contributes to function. The superfamily includes 5'-deoxynucleotidase YfbR found in bacteria and fungi. The *E. coli* YfbR HD-domain functions as a 5'-nucleotidase selectively functioning on deoxynucleotides [5]. We identified a single representative HD-domain sequence in the *T. brucei* genome specific to the 5'-deoxynucleotidase sequence signature (Pfam13023: HD_3) corresponding to a hypothetical protein encoded by the Tb09.211.2190 gene. The *T. brucei* HD-domain-containing sequence also recognizes the YfbR family with significant scores (E-value 2e-36), suggesting that it could function similarly as a 5'-deoxynucleotidase in pyrimidine metabolism.

1. Rigden DJ. The histidine phosphatase superfamily: structure and function. Biochem J. 2008;409(2):333-48. doi: 10.1042/BJ20071097. PMID: 18092946.
2. Zylka MJ, Sowa NA, Taylor-Blake B, Twomey MA, Herrala A, Voikar V, et al. Prostatic acid phosphatase is an ectonucleotidase and suppresses pain by generating adenosine. Neuron. 2008;60(1):111-22. doi: 10.1016/j.neuron.2008.08.024. PMID: 18940592; PMCID: PMC2629077.
3. Knapp K, Zebisch M, Pippel J, El-Tayeb A, Muller CE, Strater N. Crystal structure of the human ecto-5'-nucleotidase (CD73): insights into the regulation of purinergic signaling. Structure. 2012;20(12):2161-73. doi: 10.1016/j.str.2012.10.001. PMID: 23142347.
4. Kuznetsova E, Proudfoot M, Gonzalez CF, Brown G, Omelchenko MV, Borozan I, et al. Genome-wide analysis of substrate specificities of the Escherichia coli haloacid dehalogenase-like phosphatase family. The Journal of biological chemistry. 2006;281(47):36149-61. doi: 10.1074/jbc.M605449200. PMID: 16990279.
5. Zimmerman MD, Proudfoot M, Yakunin A, Minor W. Structural insight into the mechanism of substrate specificity and catalytic activity of an HD-domain phosphohydrolase: the 5'-deoxyribonucleotidase YfbR from Escherichia coli. J Mol Biol. 2008;378(1):215-26. doi: 10.1016/j.jmb.2008.02.036. PMID: 18353368; PMCID: PMC2504004.
