# Supplementary material for: Pyrimidine Salvage Enzymes Are Essential for De Novo Biosynthesis of Deoxypyrimidine Nucleotides in Trypanosoma brucei
Source: PLoS Pathog. 2016 Nov 7;12(11):e1006010. doi: 10.1371/journal.ppat.1006010 (PMC5098729; doi:10.1371/journal.ppat.1006010)
Supplement: S1 Table — (PDF) [file ppat.1006010.s003.pdf]

S1 Table. ***T. brucei*** **sequences with identified 5'nucleotidase signatures**

Accession	Annotation	Tb gene locus	CDD ID	E-value	Fold group
XP_844652.1	serine/threonine protein phosphatase PP1	Tb927.4.5030	249630	3.0E-31	metallophosphatase
XP_847568.1	serine/threonine protein phosphatase PP1	Tb927.8.7390	249630	4.0E-31	metallophosphatase
XP_828883.1	serine/threonine protein phosphatase catalytic subunit	Tb11.01.0450	249630	4.0E-29	metallophosphatase
XP_843718.1	serine/threonine-protein phosphatase	Tb927.3.1240	249630	5.0E-28	metallophosphatase
XP_829256.1	protein phosphatase 4 catalytic subunit	Tb11.01.3770	249630	6.0E-27	metallophosphatase
XP_844512.1	serine/threonine-protein phosphatase PP1	Tb927.4.3620	249630	2.0E-25	metallophosphatase
XP_844513.1	serine/threonine-protein phosphatase PP1	Tb927.4.3630	249630	2.0E-25	metallophosphatase
XP_844514.1	serine/threonine-protein phosphatase PP1	Tb927.4.3640	249630	2.0E-25	metallophosphatase
XP_829753.1	protein phosphatase 2A catalytic subunit	Tb11.01.8740	249630	3.0E-25	metallophosphatase
XP_844506.1	serine/threonine protein phosphatase PP1	Tb927.4.3560	249630	1.0E-24	metallophosphatase
XP_845108.1	serine/threonine protein phosphatase	Tb927.5.4380	249630	2.0E-22	metallophosphatase
XP_827850.1	serine/threonine protein phosphatase type 5	Tb10.05.0110	249630	2.0E-22	metallophosphatase
XP_844511.1	serine/threonine-protein phosphatase PP1	Tb927.4.3610	249630	2.0E-22	metallophosphatase
XP_001219099.1	Ser/Thr protein phosphatase	Tb927.1.4050	249630	6.0E-22	metallophosphatase
XP_829310.1	serine/threonine protein phosphatase	Tb11.01.4320	249630	2.0E-21	metallophosphatase
XP_803443.1	serine/threonine protein phosphatase	Tb09.160.0480	249630	1.0E-20	metallophosphatase
XP_844337.1	serine/threonine protein phosphatase	Tb927.4.1870	249630	2.0E-20	metallophosphatase
XP_822896.1	serine/threonine protein phosphatase	Tb10.70.0250	249630	6.0E-18	metallophosphatase

XP_846950.1	hypothetical protein	Tb927.8.1130	249630	7.0E-18	metallophosphatase
XP_828574.1	protein phosphatase	Tb11.02.2630	249630	1.0E-17	metallophosphatase
XP_951632.1	DNA repair protein	Tb927.2.4390	249630	6.0E-16	metallophosphatase
XP_822888.1	serine/threonine protein phosphatase 2b catalytic subunit A2	Tb10.70.0350	249630	2.0E-15	metallophosphatase
XP_845187.1	serine/threonine protein phosphatase	Tb927.6.640	249630	3.0E-13	metallophosphatase
XP_844583.1	serine/threonine protein phosphatase	Tb927.4.4330	249630	6.0E-12	metallophosphatase
XP_847629.1	diadenosine tetraphosphatase	Tb927.8.8040	249630	5.0E-11	metallophosphatase
XP_845580.1	serine/threonine protein phosphatase	Tb927.6.4630	249630	3.0E-10	metallophosphatase
XP_844887.1	hypothetical protein	Tb927.5.2130	249630	5.0E-09	metallophosphatase
XP_845198.1	serine/threonine protein phosphatase	Tb927.6.750	249630	3.0E-08	metallophosphatase
XP_823252.1	hypothetical protein	Tb10.406.0620	249630	1.0E-04	metallophosphatase
XP_001218808.1	hypothetical protein	Tb927.1.1050	249630	2.0E-04	metallophosphatase
XP_844261.1	hypothetical protein	Tb927.4.1110	249630	1.0E-03	metallophosphatase
XP_845245.1	hypothetical protein	Tb927.6.1230	249630	2.0E-03	metallophosphatase
XP_829485.1	vacuolar sorting protein	Tb11.01.5900	249630	2.0E-03	metallophosphatase
XP_829370.1	membrane-bound acid phosphatase 1 precursor	Tb11.01.4701	249773	4.0E-38	Histidine Phosphatase
XP_822949.1	membrane-bound acid phosphatase 2	Tb10.6k15.3560	249773	3.0E-31	Histidine Phosphatase
XP_822937.1	acid phosphatase	Tb10.6k15.3720	249773	7.0E-24	Histidine Phosphatase
XP_829237.1	membrane-bound acid phosphatase	Tb11.01.3610	249773	1.0E-08	Histidine Phosphatase
XP_828374.1	glycerolphosphate mutase	Tb11.02.0440	249773	2.0E-06	Histidine Phosphatase
XP_829098.1	phosphoglycerate mutase-like protein	Tb11.01.2110	249773	3.0E-04	Histidine Phosphatase
XP_828373.1	hypothetical protein	Tb11.02.0430	249773	6.0E-04	Histidine Phosphatase
XP_822699.1	fructose-6-phosphate2-kinase	Tb10.70.2700	249773	4.0E-03	Histidine Phosphatase
XP_828515.1	hypothetical protein	Tb11.02.1940	249773	4.0E-03	Histidine Phosphatase

XP_829590.1	hypothetical protein	Tb11.v4.0008	147785	6.0E-03	HAD domain-related
XP_847580.1	p-nitrophenylphosphatase	Tb927.8.7510	257673	2.0E-23	HAD domain-related
XP_827334.1	hypothetical protein	Tb09.211.1880	257673	5.0E-23	HAD domain-related
XP_828918.1	haloacid dehalogenase-like hydrolase	Tb11.01.0120	257673	6.0E-05	HAD domain-related
XP_828367.1	hypothetical protein	Tb11.02.0360	257673	1.0E-03	HAD domain-related
XP_823441.1	hypothetical protein	Tb10.389.1370	257673	3.0E-03	HAD domain-related
XP_844391.1	hypothetical protein	Tb927.4.2410	257744	5.0E-08	HAD domain-related
XP_829616.1	hypothetical protein	Tb11.01.7230	257744	1.0E-07	HAD domain-related
XP_827907.1	hypothetical protein	Tb10.61.2520	257744	2.0E-05	HAD domain-related
XP_846144.1	hypothetical protein	Tb927.7.5210	257744	4.0E-04	HAD domain-related
XP_845010.1	calcium-translocating P-type ATPase	Tb927.5.3400	257744	5.0E-04	HAD domain-related
XP_823353.1	hypothetical protein	Tb10.26.0480	257744	8.0E-04	HAD domain-related
XP_822666.1	hypothetical protein	Tb10.70.3070	257744	2.0E-03	HAD domain-related
XP_822886.1	phosphomannomutase	Tb10.70.0370	257744	3.0E-03	HAD domain-related
XP_829314.1	hypothetical protein	Tb11.01.4360	257744	3.0E-03	HAD domain-related
XP_828217.1	copper-transporting ATPase-like protein	Tb11.47.0023	257744	6.0E-03	HAD domain-related
XP_827362.1	hypothetical protein	Tb09.211.2190	257465	3.0E-38	HD-domain*

*Note: the homologs of Tb09.211.2190 (HD-domain) are present in *T. cruzi*: (XP_821610.1) Tc00.1047053508461.150 and *L. donovani*: (XP_003864995.1) LDBPK_354170.
