# Supplementary material for: Pyrimidine Salvage Enzymes Are Essential for De Novo Biosynthesis of Deoxypyrimidine Nucleotides in Trypanosoma brucei
Source: PLoS Pathog. 2016 Nov 7;12(11):e1006010. doi: 10.1371/journal.ppat.1006010 (PMC5098729; doi:10.1371/journal.ppat.1006010)
Supplement: S2 Table — List of cloning primers and corresponding sequences (5’-3’) used throughout the study. The primer names contain information regarding their target, restriction sites, and functions. (PDF) [file ppat.1006010.s004.pdf]

S2 Table S2. PCR and cloning primers.

#	Primer name	Sequence (5' to 3')
1	*Tb* TK 5' UTR forward	cggaagagttctttgtgctttgtag
2	*Tb* TK 3' UTR reverse	ccttcaggttcgtgtgaatgttcttc
3	*Tb* TK 5' UTR reverse (HYG)	ggtgagttcaggctttttcatttgcaatgttgagatgttggtgattcctgtcg
4	*Tb* TK 3' UTR forward (HYG)	gtccgagggcaaaggaatagtgccaggtgtactattgcgcaacg
5	*Tb* TK 5' UTR reverse (PAC)	gtgggcttgtactcggtcatttgcaatgttgagatgttgg
6	*Tb* TK 3' UTR forward (PAC)	gacccgcaagcccggtgcctgatgccaggtgtactattgc
7	*Tb* TK fusion forward	ctaactgagaagctcatcctttc
8	*Tb* TK fusion reverse	ccaaaggtgtacggctacgataagg
9	HYG forward	atgaaaaagcctgaactcacc
10	HYG reverse	ctattcctttgccctcggac
11	PAC forward	atgaccgagtacaagcccac
12	PAC reverse	tcaggcaccgggcttgcgggt c
13	*Tb* TK HindIII NT-FLAG forward	gctaaagcttatggactacaaagacgacgacgacaaacacgacggagatggcaat
14	*Tb* TK BamHI reverse	tagcggatccctaagtagtatcaacggccatttg
15	*Tb* TK 3' UTR RNAi forward	ttggttcgttacttttcccgagg
16	*Tb* TK 3' UTR RNAi reverse	accttgacaagcgtttcaccac
17	*Tb* TK ORF qPCR reverse	acatgtacttggctgtgtgc
18	*Tb* TK ORF qPCR reverse	atgtcttctgcgcatcaacg
19	*Tb* TK HindIII forward	gctaaagcttatgcacgacggagatggcaat
20	*Tb* TK BamHI AU1 reverse	tagcggatcctcatttgtcgtcgtcgtctttgtagtcagtagtatcaacggccatttg
21	*Hs* TK HindIII forward	gctaaagcttatgtcgtgtatcaatctcccaac
22	*Hs* TK BamHI FLAG reverse	tagcggatccctatttgtcgtcgtcgtctttgtagtcgtttgctgggctacactggag
23	*Tb* TK E286A forward	gtgatcgcggtggatgccggccaatttttttcctgg
24	*Tb* TK E286A reverse	ccaggaaaaaaattggccggcatccaccgcgatcac
25	*Hs* TK K32I forward	ccaatgttttccggcatctctaccgaacttatgcg
26	*Hs* TK K32 reverse	cgcataagttcggtagagatgccggaaaacattgg
27	*Hsv* TK forward	gctaaagcttatggcctcgtaccccggcca tc
28	*Hsv* TK reverse	tagcggatcctcagttagcctcccccatctcccgggcaaac
29	CDA 5' UTR forward	gaggcgcaaataggctggttgtgc
30	CDA 3' UTR reverse	cggtataataagcagcagcaaaag
31	CDA 5' UTR reverse (HYG)	ggtgagttcaggctttttcattgtgacgcgatgcttctccc
32	CDA 3' UTR forward (HYG)	gtccgagggcaaaggaataggtaaacggtttctgagggggcg
33	CDA 5' UTR reverse (PAC)	gtgggtttatactcggtcattgtgacgcgatgcttctccc
34	CDA 3' UTR forward (PAC)	gactcgaaagccaggtgcctaagtaaacggtttctgagggggcg
35	PAC (optimized) forward	atgaccgagtataaacccacag
36	PAC (optimized) reverse	ttaggcacctggctttcgagtc
37	CDA fusion forward	gcgaatacgcgaaaggttatggg
38	CDA fusion reverse	gaatcgcgggaaagttgctgcgg
39	CDA ORF qPCR forward	tgaagaccggttgcttgaag
40	CDA ORF qPCR reverse	tggacagtaacttcgctggtg
41	*Hs* DCTD HindIII FLAG forward	gctaaagcttatggactacaaagacgacgacgacaaaagtgaagtttcctgcaagaaacg
42	*Hs* DCTD BamHI reverse	tagcggatccctactgcaacttttgactcggtctg
43	dTTP assay oligo 1	ttattattattattattaggcggtggaggcgg
44	dTTP assay oligo 2	ccgcctccaccgcc
45	Nucleotidase BsaI forward	gcggtctcaaggtatggacaccatcgaattcctgc
46	Nucleotidase XbaI reverse	gctctagattagctcttacggctcggacggg
