# Supplementary figures and images for: Pyrimidine Salvage Enzymes Are Essential for De Novo Biosynthesis of Deoxypyrimidine Nucleotides in Trypanosoma brucei
Source: PLoS Pathog. 2016 Nov 7;12(11):e1006010. doi: 10.1371/journal.ppat.1006010 (PMC5098729; doi:10.1371/journal.ppat.1006010)

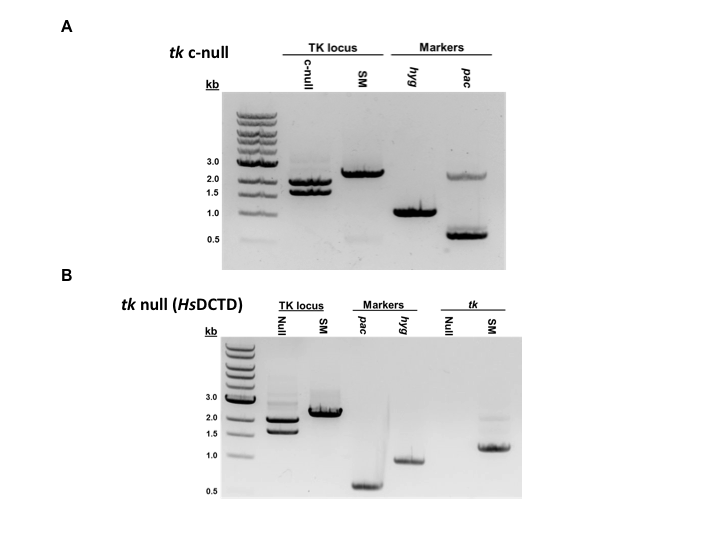

Supplement: S1 Fig — Amplification of the TK locus using primers flanking the TK 5’ and 3’ UTRs (A,B), selectable markers(A,B), and TK ORF (B). The TK locus was amplified from genomic DNA extracted from TK c-null and WT SM cells and the selectable markers were amplified from TK c-null genomic DNA. The TK gene was amplified from both the WT SM and human DCTD expressing TK null genomic DNA. (TIFF) [file ppat.1006010.s005.tiff]

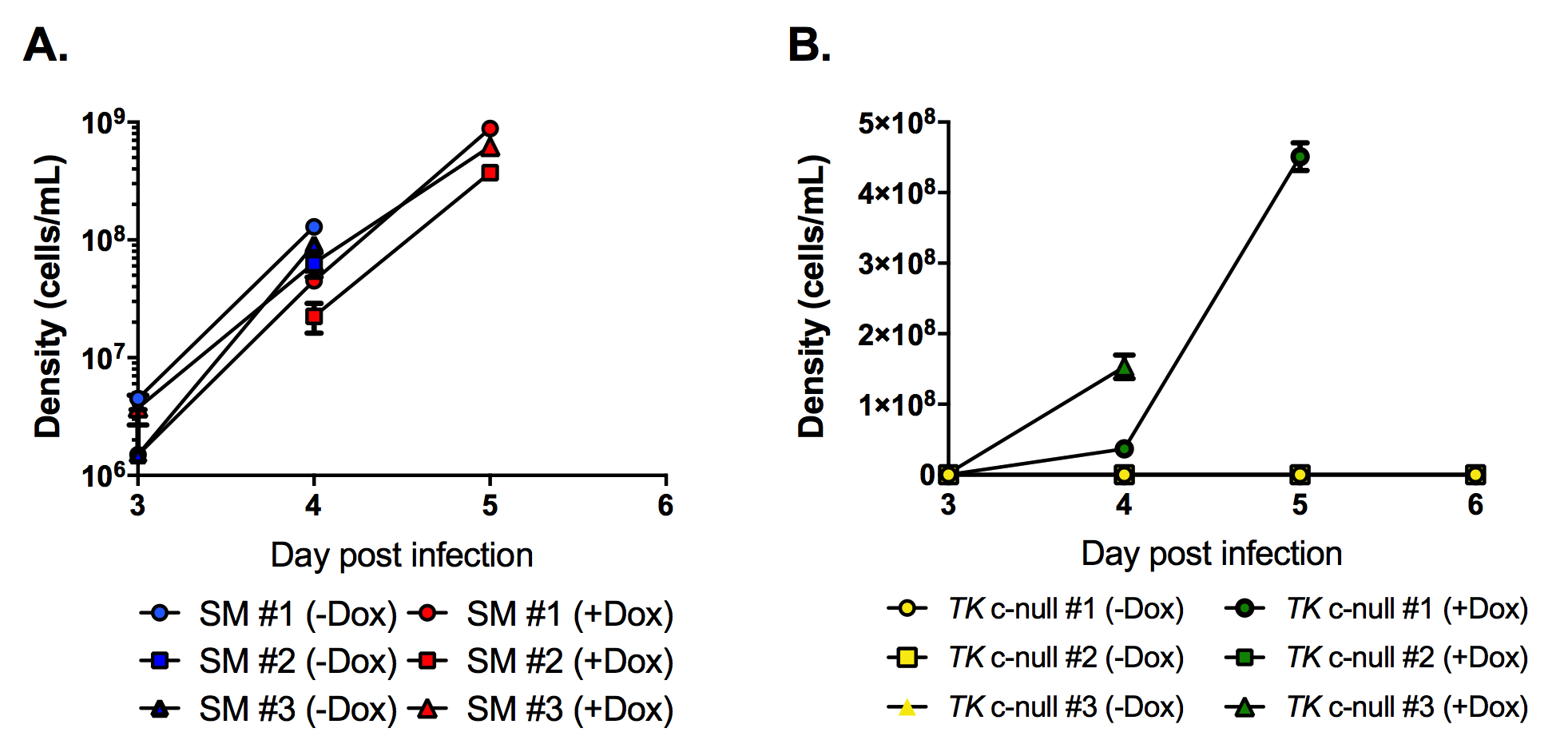

Supplement: S2 Fig — Graphs show parasitemia levels (cells/ml) in blood collected the first 6 days post infection for three mice per each group. A. SM cells ±Dox. B. TK c-null cells ±Dox. Mouse #2 in the TK c-null +Dox arm was delayed in the course of infection and did not succumb to parasitemia until day 18. Parasites were not observed at any time over the 30 days in the TK c-null -Dox treated animals. (TIFF) [file ppat.1006010.s006.tiff]

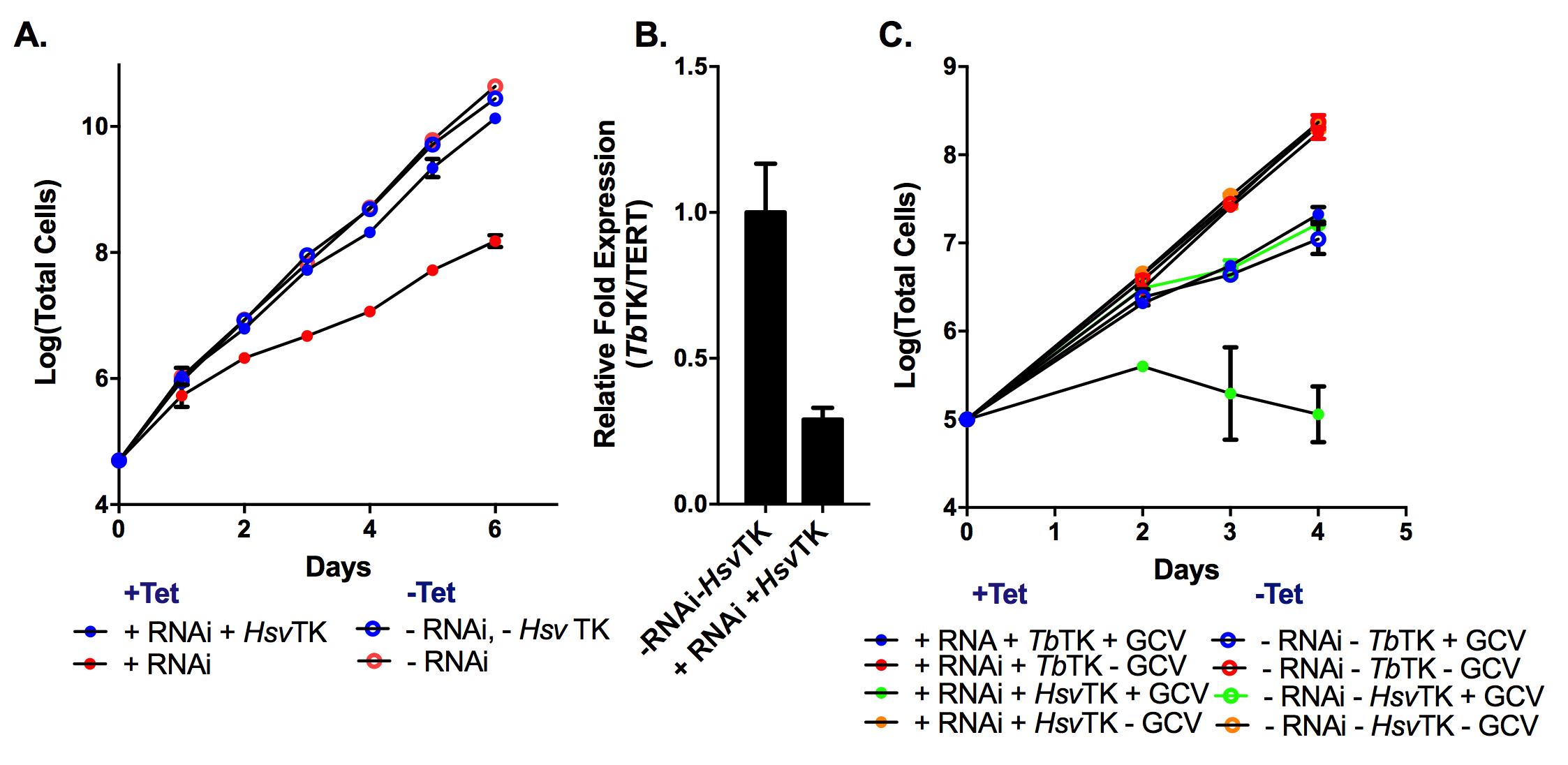

Supplement: S3 Fig — A TK RNAi cells (±Tet) expressing Tet regulated HsvTK grown in parallel with the TK RNAi control (±Tet). B. Growth analysis of TK RNAi cells (±Tet) expressing Tet regulated TbTK or HsvTK grown in the presence or absence of ganciclovir (GCV-50 μg/ml). C. qPCR analysis of relative TK expression in HsvTK rescue cells 48 h after Tet supplementation compared to SM (WT) cells. TbTK expression is normalized with TERT expression and error bars represent standard error of the mean (SEM) calculated from triplicate data. All growth experiments were performed in HMI-19 media and the error bars represent the standard deviation (SD) for biological triplicates. (TIFF) [file ppat.1006010.s007.tiff]

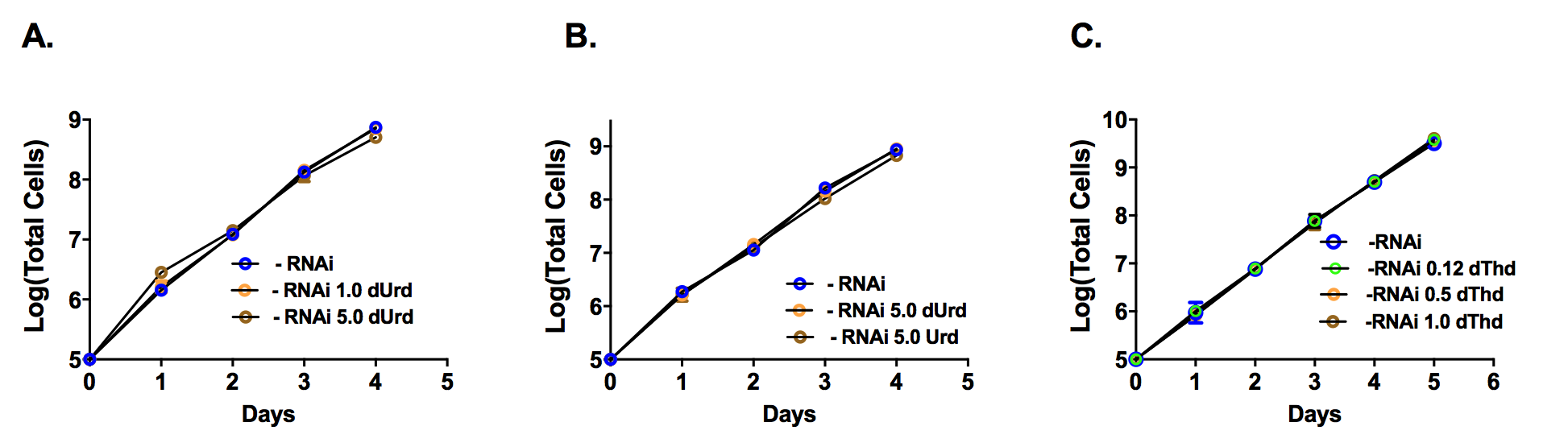

Supplement: S5 Fig — Control studies for data reported in Fig 4 to demonstrate that exogenous nucleosides do not affect growth of cells that are expressing TK. A. Cells were grown in normal serum based media plus 0, 1 or 5 mM dUrd in the absence of Tet. B. Cells were grown plus or minus 5 mM dUrd or Urd. C. Cells were grown plus 0, 0.12, 0.5 or 1.0 mM dThd. Error bars represent the standard deviation (SD) for biological triplicates. (TIFF) [file ppat.1006010.s009.tiff]

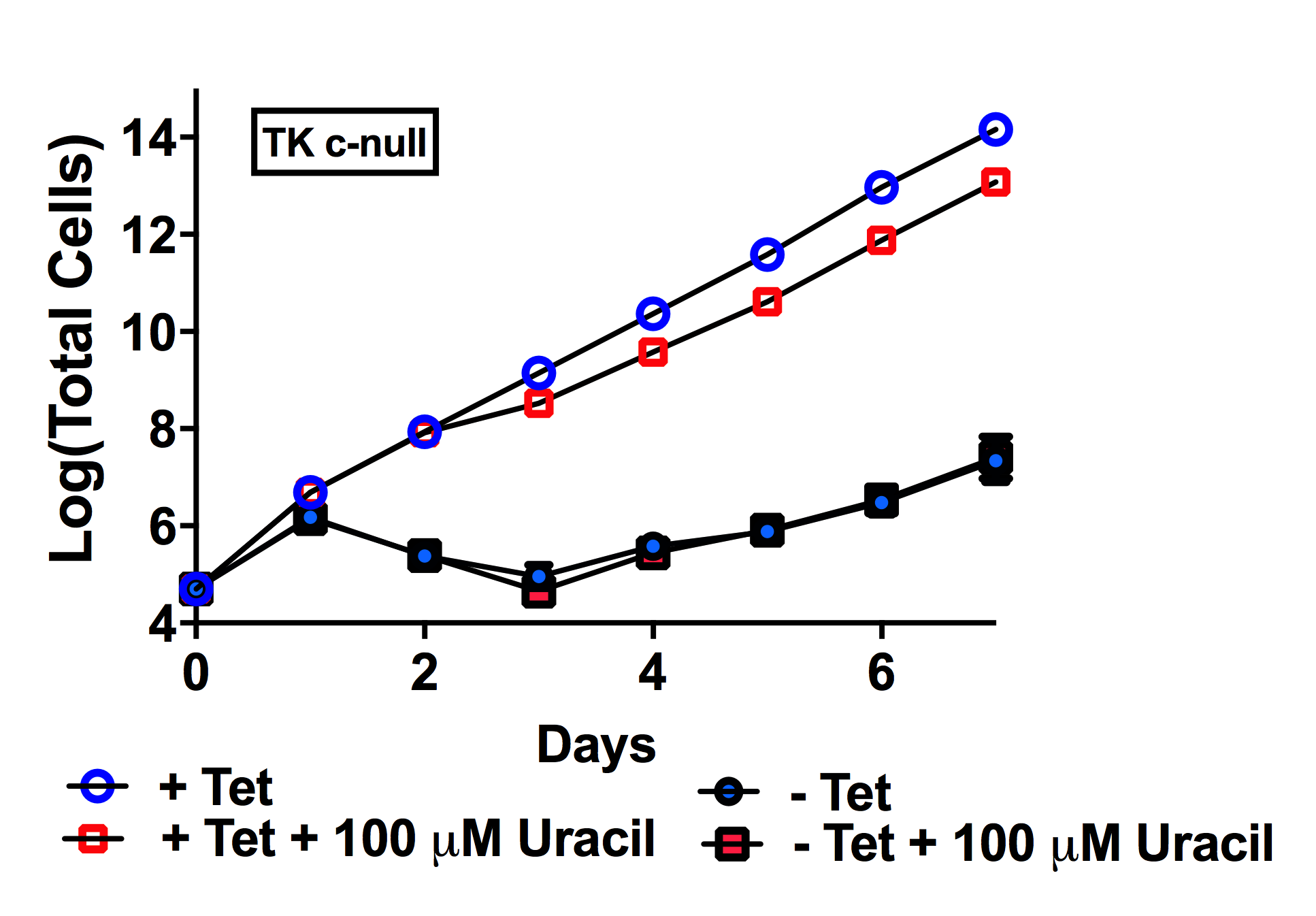

Supplement: S6 Fig — A. Growth analysis of TK c-null cells (±Tet) in HMI-19 media supplemented with uracil (μM). Error bars represent the standard deviation (SD) for biological triplicates. (TIFF) [file ppat.1006010.s010.tiff]

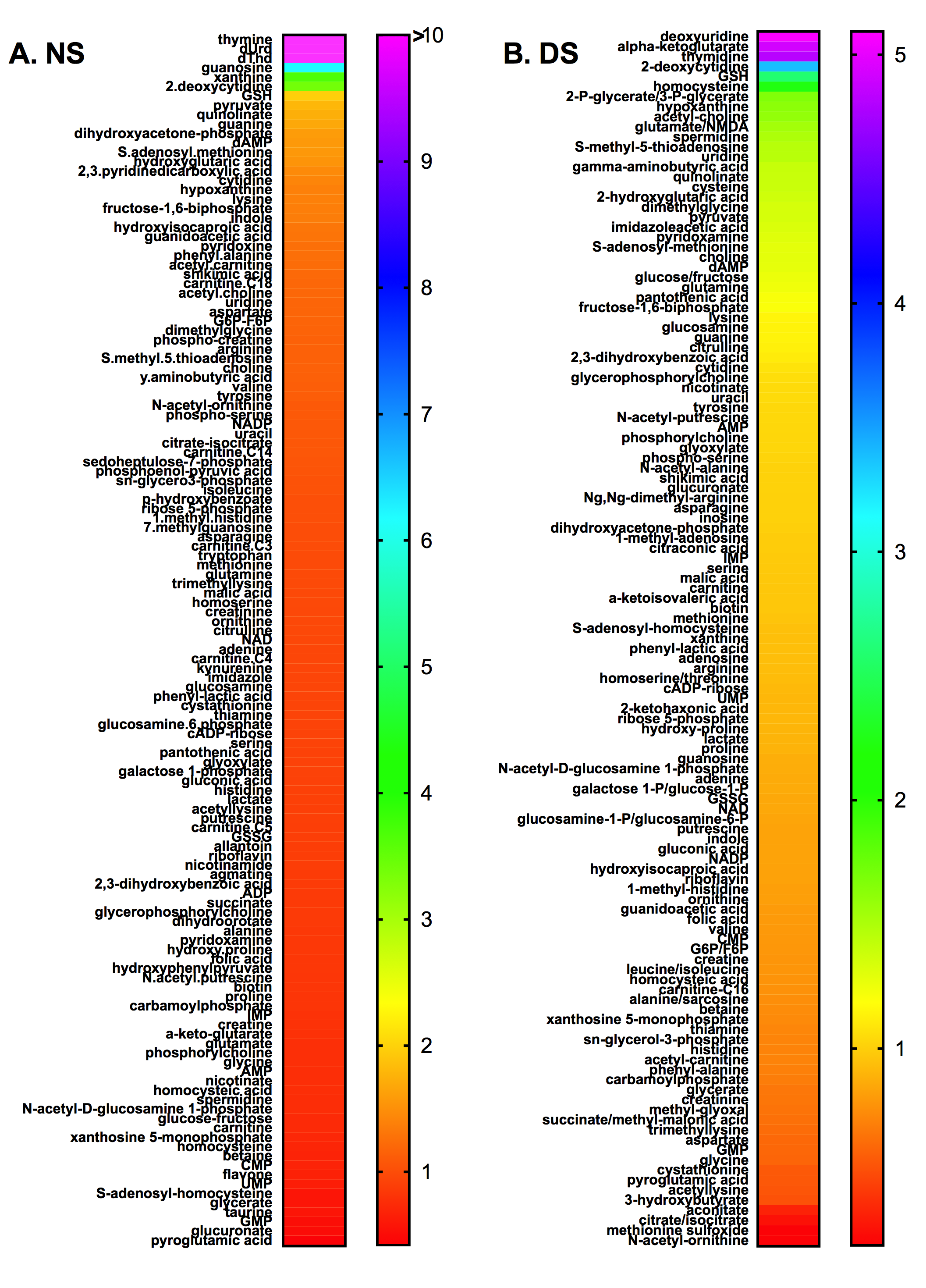

Supplement: S7 Fig — A-B. Heat maps represent average fold changes (-Tet 24hr/+Tet) in relative metabolite abundance. A) normal serum (NS); B) dialyzed serum (DS). For NS, pink indicates fold changes exceeding the scale (fold change > 10). (TIFF) [file ppat.1006010.s011.tiff]

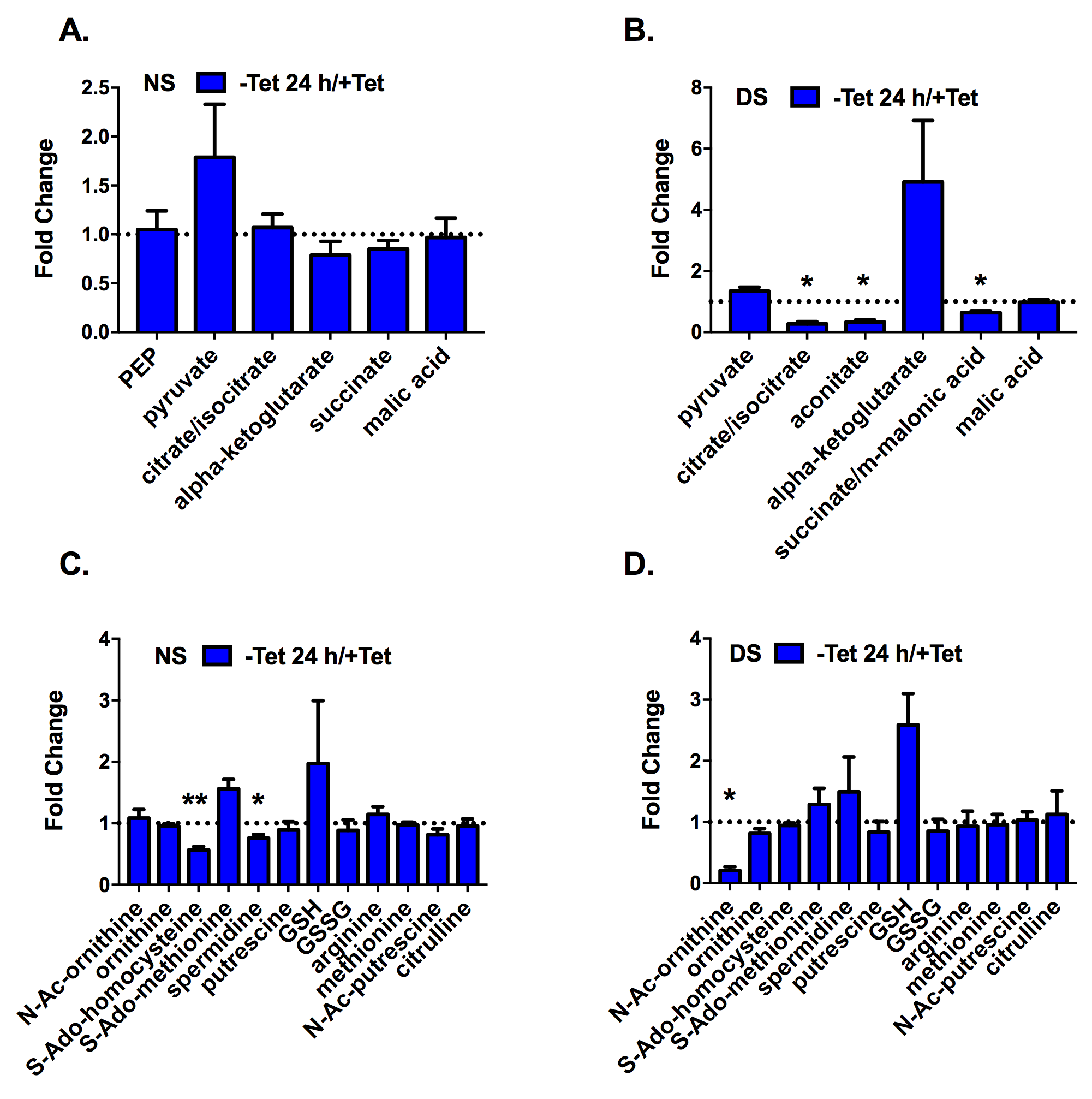

Supplement: S8 Fig — Fold change (-Tet 24h/+Tet) for A, B) TCA cycle metabolites NS vs DS or for C, D) polyamines NS vs DS. Error bars represent SEM calculated from biological triplicate data. Metabolites that showed a significant difference between the conditions are marked * P<0.05, ** P<0.01. Statistical significance was calculated as described in Fig 6 of the main paper. (TIFF) [file ppat.1006010.s012.tiff]

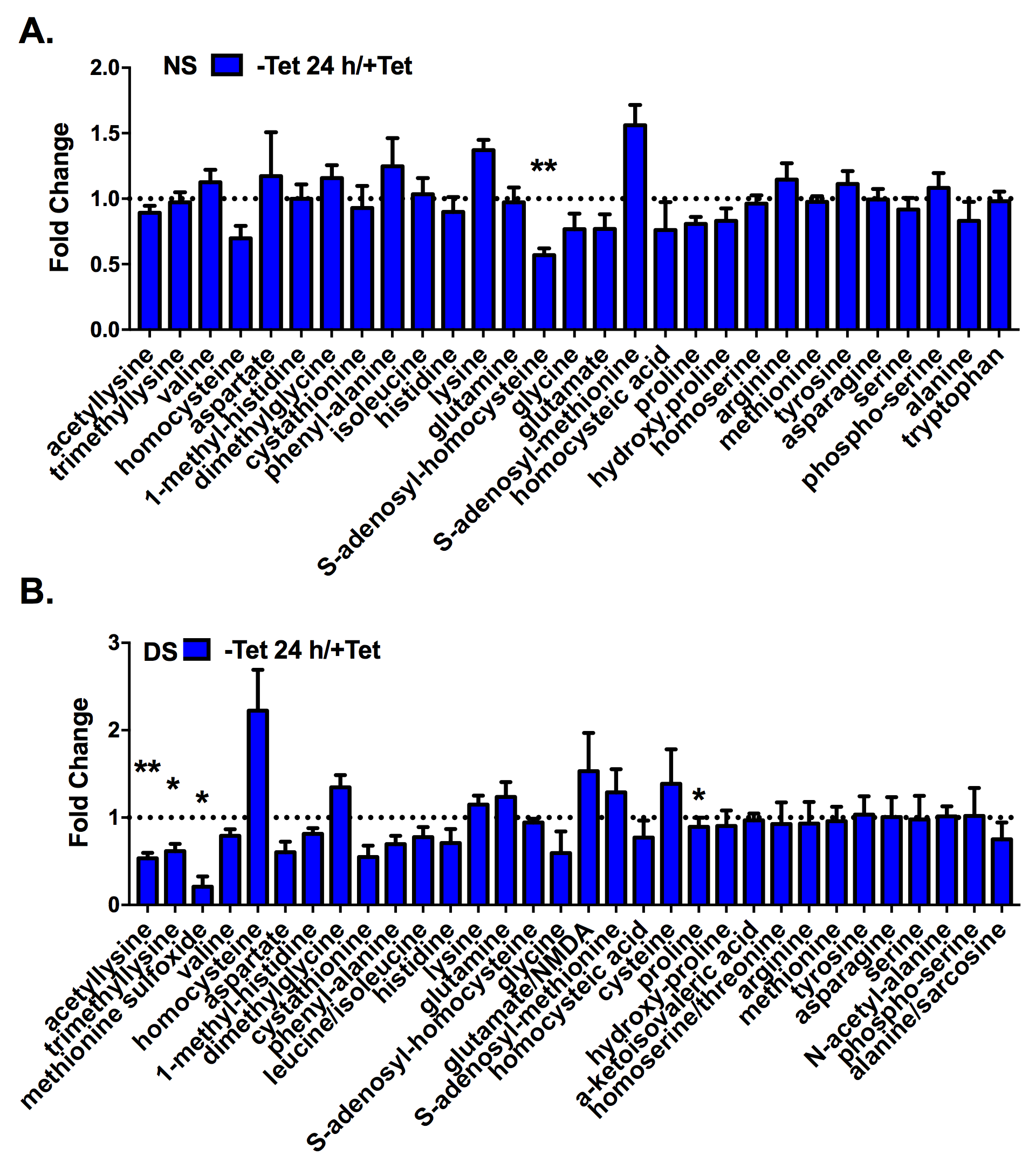

Supplement: S9 Fig — A-B. Fold change (-Tet 24h/+Tet) in relative abundance of amino acids and related metabolites grown in NS and DS. Error bars represent SEM calculated from biological triplicate data and statistical analysis is as described in Fig 6 and S7 Fig. (TIFF) [file ppat.1006010.s013.tiff]

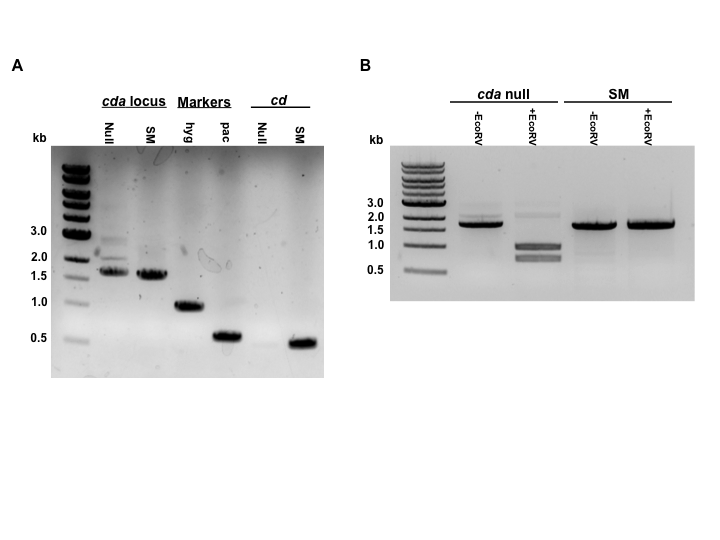

Supplement: S10 Fig — A. Amplification of the CDA locus using primers flanking the CDA 5’ and 3’ UTRs, selectable markers, and CDA ORF. The CDA locus was amplified from genomic DNA extracted from CDA null and SM cells. The selectable markers were amplified from CDA null genomic DNA. The CDA gene was amplified from both SM and CDA null genomic DNA. B. The puromycin selection marker and CDA gene possessed similar sized PCR products. The PCR product corresponding to the amplified puromycin resistance gene contained a unique EcoRV restriction site, allowing for discrimination between the two PCR products. (TIFF) [file ppat.1006010.s014.tiff]

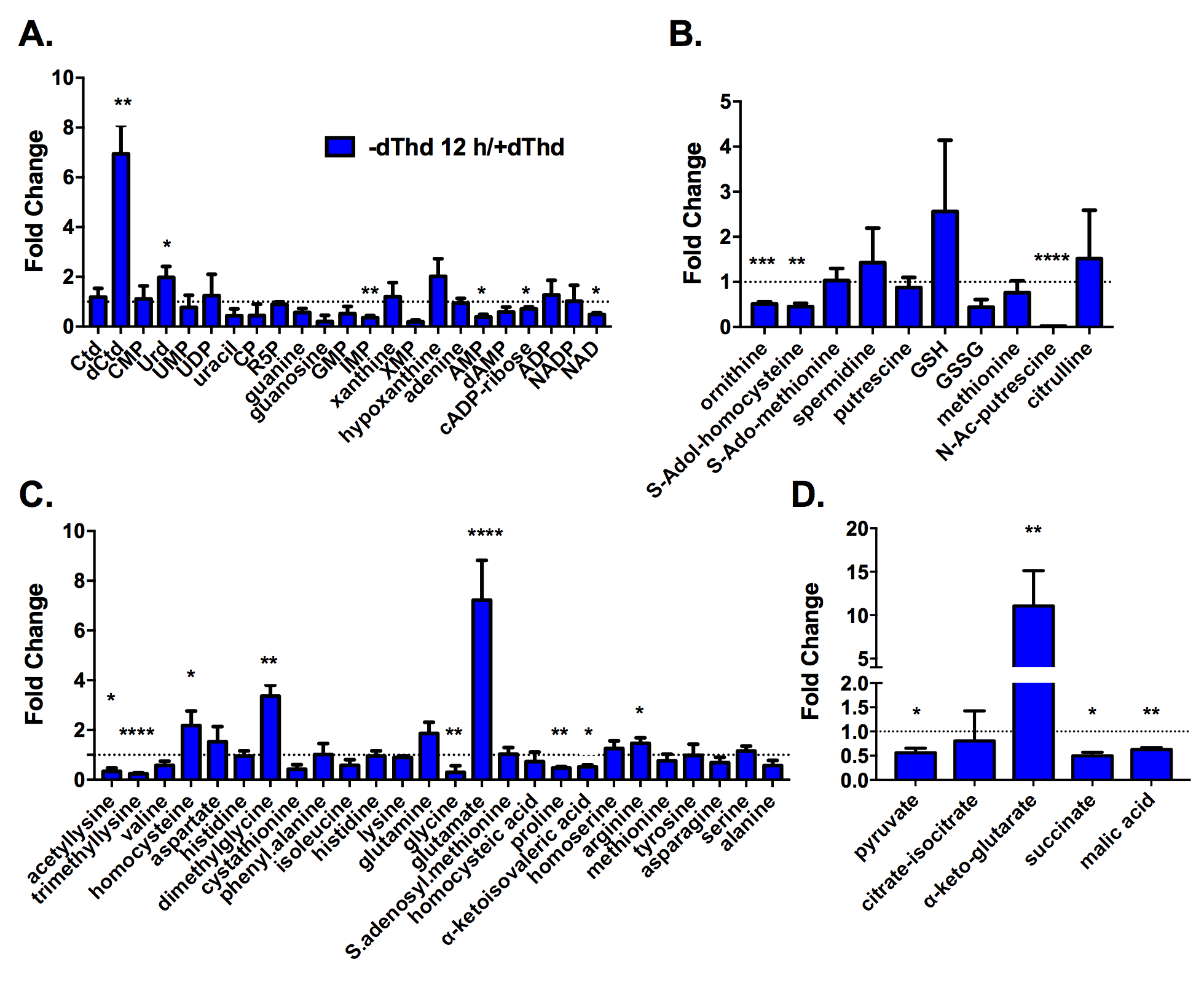

Supplement: S11 Fig — Fold change in relative metabolite abundance comparing CDA null cells (-Thd 12h/+Thd) grown in media containing dialyzed FBS in the absence of Thd for 12 h versus cells grown in media supplemented with 0.5 mM Thd. Panels represent A) nucleotides B) polyamines C) amino acids and D) TCA cycle intermediates. Error bars represent SEM calculated from biological triplicate data. Metabolites that showed a significant difference between the conditions are marked * P<0.05, ** P<0.01. Statistical significance was calculated as described in Fig 6. (TIFF) [file ppat.1006010.s015.tiff]

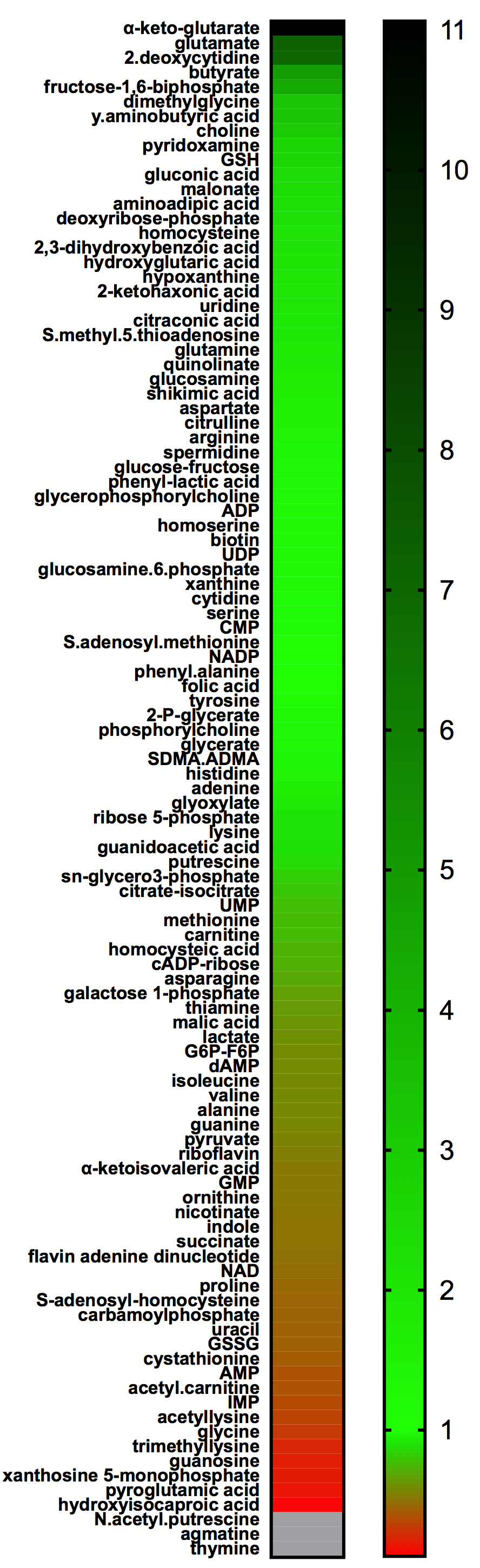

Supplement: S12 Fig — Heat map represent average fold changes (-Thd 12hr/+Thd) in relative metabolite abundance. Cells colored gray represent metabolites with fold decreases greater than 10. The presence of thymine in the +Thd treated cells is likely caused by contamination of the commercial Thd source with thymine. (TIFF) [file ppat.1006010.s016.tiff]

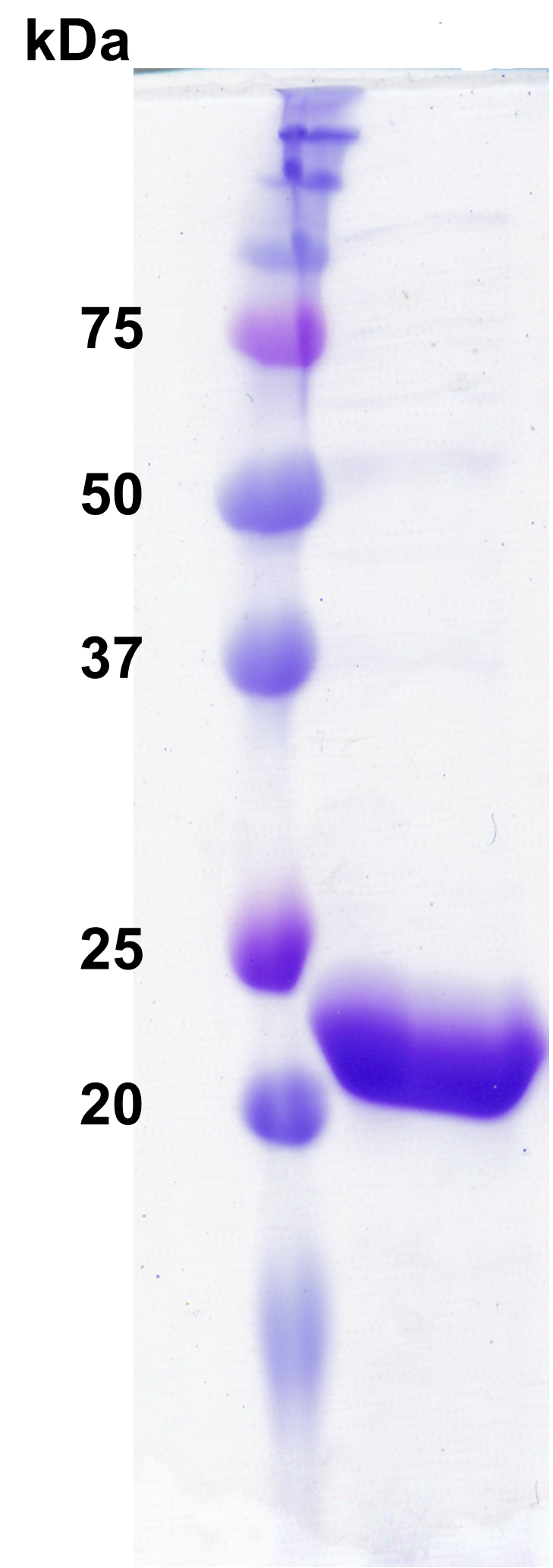

Supplement: S14 Fig — The gel shows the final purified 5’-nucleotidase (lane 2) after cleavage and removal of the His6-Sumo tag. 10 μg of 5’-nucleotidase was loaded on the gel. The molecular weight of protein standards in lane 1 are shown. (TIF) [file ppat.1006010.s018.tif]
