# Supplementary material for: Pyrimidine Salvage Enzymes Are Essential for De Novo Biosynthesis of Deoxypyrimidine Nucleotides in Trypanosoma brucei
Source: PLoS Pathog. 2016 Nov 7;12(11):e1006010. doi: 10.1371/journal.ppat.1006010 (PMC5098729; doi:10.1371/journal.ppat.1006010)
Supplement: S4 Fig — E. coli (Ec-P23331), Humans (Hs – P04183), Mus muscaris (Mm – P04184), Leishmania donovani (Ld – Q4QC75), inactive N-terminal TbTK domain, and active C-terminal TbTK domain. Residues highlighted in green represent highly conserved residues in active TKs selected for the mutagenesis studies (TbTK E286A and HsTK K32I). (PDF) [file ppat.1006010.s008.pdf]

```
EcTK            -----------------MAQLYFYYSAMNAGKSTALLQSSYNYQERGMRTVVYTAEIDDR   43
HsTK            MSCINLPTVLPGSPSKTRGQIQVILGPMFSGKSTELMRRVRRFQIAQYKCLVIKYAKDTR   60
MmTK            MSYINLPTVLPSSPSKTRGQIQVILGPMFSGKSTELMRRVRRFQIAQYKCLVIKYAKDTR   60
LdTK            ---------------MFRGRIELIIGPMFAGKTTELMRRVKREIHARRSCFVIKYSKDTR   45
NT-TbTK         -------------MHDGDGNIELIIGPMFAGKTTELMRRVQRHKHAQRSCYIINYSRN-S   46
CT-TbTK         ------------VPNGAHGRIELIIGPMFAGKTTELMRRVQRHKHAQRSCYIIKYTGDTR   48
                                  ..: .  . * :**:* *::   .         : .   :

EcTK            FGAGKVSSRIGLSSPAKLFNQNSSLFDEIRAEHEQQAIHCVLVDECQFLTRQQVYELSEV   103
HsTK            YSSSF-CTHDRNTMEA-L---PACLLRDVA--QEALGVAVIGIDEGQFFPDIV--EFCEA   111
MmTK            YSNSF-STHDRNTMDA-L---PACMLRDVT--QESLGVAVIGIDEGQFFPDIV--DFCEM   111
LdTK            YDEHNVASHDQLMLRAQA---AVSQLTEVR--DTWKRFDVLAIDEGQFFSDLV--NFCNT   98
NT-TbTK         YQNQRLSTHDQLSLTANV---SIAKLSEVC--DEWRDYDVIAVDNGQFFPDVV--GFCAR   99
CT-TbTK         YSEGAITSHDQRALTANV---SVSNLHDVG--DEWRKYDVIAVDEGQFFPGVA--AFCSK   101
                :      ::      *       . : ::   .       : :*: **:       :.

EcTK            VDQLDIPVLCYGLRTDFRGELFIGSQYLLAWSDKLVELKTICFCGRKASMVLRLDQAGRP   163
HsTK            MANAGKTVIVAALDGTFQRKPFGAILNLVPLAESVVKLTAVCMECFREAA-----YTKRL   166
MmTK            MANEGKTVIVAALDGTFQRKAFGSILNLVPLAESVVKLTAVCMECFREAA-----YTKRL   166
LdTK            AADAGKVVMVSALDGDYRRKPFGQICELVPYCEAVDKLTAVCMMCHEQPAC----FTRRT   154
NT-TbTK         AANEGKTVIVSALDVDCRETPFDEVCRLVPRAESVLKLSAVCMECHEHDAF----LTYRT   155
CT-TbTK         AADSGKVVIVSALDADYLQEPFEEICLLVSRADSVVKLSAVCMECHNRKAS----FTYRT   157
                  :    *:  .*        *     *:  .: : :*.::*:   .         : *

EcTK            YNEGEQVVIGGNERYVSVCRKHYKEALQVDSLTAIQ------ERHRHD------------   205
HsTK            GTEKEVEVIGGADKYHSVCRLCYFKKASGQPAGPDN--------KENCPVPGK-------   211
MmTK            GLEKEVEVIGGADKYHSVCRLCYFKKSSAQTAGSDN---------KNCLVLGQ-------   210
LdTK            VNVEQQELIGGADMYIATCRECYSKQQLPSIEEMRTQQMAIKEVEKRYLGMSDKR---AT   211
NT-TbTK         IESNERELYGGADMYLAVCRWCYKQLTMSHVDAQ------------------KTSASTAA   197
CT-TbTK         VKSDERKLVGGSDMYMSVCRSCYETKRNMVQTEKYIY---------SCVGINEGSYSECS   208
                    :  : ** : * :.**  *

EcTK            ------------------------------------------------------------   205
HsTK            PGEA--VAARKLFAPQQILQCSPAN-----------------------------------   234
MmTK            PGEA--LVVRKLFASQQVLQYNSAN-----------------------------------   233
LdTK            AGPQTPEKPAGGWGTKTGVATLPTMATEGAASSGASAGMKSGRDLCEVQTFTTEAPKYQR   271
NT-TbTK         ------VVPNGAHG----------------------------------------------   205
CT-TbTK         PGPS-ERSSAGTSGVQTSVKVDEQNCTEPNT-EAKKMPLKRKRNQMAVDTT---------   257

EcTK            -------------   205
HsTK            -------------   234
MmTK            -------------   233
LdTK            VEPACTASAASSE   284
NT-TbTK         -------------   205
CT-TbTK         -------------   257
```
