# Supplementary material for: Pyrimidine Salvage Enzymes Are Essential for De Novo Biosynthesis of Deoxypyrimidine Nucleotides in Trypanosoma brucei
Source: PLoS Pathog. 2016 Nov 7;12(11):e1006010. doi: 10.1371/journal.ppat.1006010 (PMC5098729; doi:10.1371/journal.ppat.1006010)
Supplement: S13 Fig — Sequences closest to the protein encoded by T. brucei Tb09.211.2190 were collected by BLAST against the RefSeq database, with HDDC2 being the closest representative in human. We generated a multiple sequence alignment with PROMALS-3D using the structures of human HDDC2 (4dmb), E.coli YfbR (2pau), M. magnetotacticum (3kh1), P. furiosus (1xx7), and A. fulgidus (1ynb); together with select HDDC2 homologs defined by HomoloGene and representative protists close to T.brucei. The HDDC2/YfbR sequences are ubiquitous, with representatives all three domains of life. Representatives are labeled to the left by PDB ID or NCBI accession, followed by species, and colored according to taxonomy: bacteria (blue labels), archaea (red labels), and eukaryota (animals black, fungi orange, plants green, and protists magenta). Secondary structures are indicated above the alignment, with H representing helix. Active site residues defined in YfbR (2pau) are invariant and are labeled above the alignment, including metal coordinating residues (H), nucleotide phosphate binding (P), nucleotide ribose binding, (R), and catalytic (C). Residue positions are highlighted according to conservation: including mainly hydrophobic (yellow) and small (gray) positions that dictate structure, and mainly polar (black) positions that dictate function. The YfbR structure (2pau) was of the E72A mutant enzyme so the alignment shows the residue as an Ala (red) even though the wild-type enzyme contains a Glu at this position. (PDF) [file ppat.1006010.s017.pdf]

```
                                                                           P                 M
SS                                                            HHHHHHHHHH    HHHHHHHH      HHHHHHHHHHHHHHHHH      HHHHHHHHH
1xx7_A       Pyrococcus furiosus                            1 SIDLILLAGKLKRIPRMGWLIKGVPNPESVADHSYRVAFITLLLAE[6]EIDVEKALKIAI
1ynb_A       Archaeoglobus fulgidus                         4 VVKFIHEVGSLKLTPRSGWLKLGIRLPESVAEHNFRAAIIAFILAL[5]VEKACKAATAAL
3kh1_A       Magnetospirillum magnetotacticum              12 QMSFVVEIDKLKTILRQTLLTD-SSRRENDAEHSWHIATMAFLLAE[4]AVQIGRVARMLL
2pau_A       Escherichia coli K-12                          1 -SHFFAHLSRLKLINR--WPLMRNVRTENVSEHSLQVAMVAHALAA[8]NVNAERIALLAM

NP_057147    Homo sapiens                                  18 LLQFLRLVGQLKRVPRTGWVYRNVQRPESVSDHMYRMAVMAMVIKD--DRLNKDRCVRLAL
NP_001264847 Gallus gallus                                 10 MLPFLRLLGQLKRVPRTGWVYRNVEKPESVSDHMYRMAVMALVTED--KSLNKDRCIRLAL
XP_002936040 Xenopus tropicalis                            14 LLQFMKLVGQLKRVPRTGWIYRQVEKPESVSDHMYRMAVMAMLTED--RKLNKDRCIRLAL
NP_001038696 Danio rerio                                    4 MLQFMKLVGQLKRVPRTGWVYRNIKQPESVSDHMYRMSMMALTIQD--ISVNKERCMKLAL
NP_609052    Drosophila melanogaster                      181 ILQFMELIGNLKHTKRTGWVLRDVNDCESISGHMYRMSMLTFLLDGS-EGLNQIRCMELAL
XP_306780    Anopheles gambiae str. PEST                    4 YIKFMEVLGNVKHLKRTGWVLRKVKDCETVSGHMYRMAMMSFFLEDS-HGLDRIRVMEMSL
NP_001256098 Caenorhabditis elegans                         6 IFELLDVLDNLKHLKRTGWVKCGVPEPETVACHMYRMAVLAMALEGQIDGLDAIRTVKMAL
NP_587821    Schizosaccharomyces pombe 972h-                9 IVPFLDCLSRLKTTPRTGWLYHGIEKPESIADHMYRMGILTMLCND--PSINKERCLKIAV
NP_009801    Saccharomyces cerevisiae S288c                49 ILAFLNVVQQLKIQRRTGYLDLGIKECESISDHMYRLSIITMLIKD--SRVNRDKCVRIAL
NP_973522    Arabidopsis thaliana                          80 AIDFLSLCTRLKTTPRAGWIKRDVKDPESIADHMYRMGLMALISSDI-PGVNRDKCMKMAI
NP_001046081 Oryza sativa Japonica Group                   53 AIDFLTLCYRLKTTKRAGWVRRGVQGPESVADHMYRMGVMALVAADLPSGVNRDRCVKMAI
CUG37041     Bodo saltans                                  10 TIEFLHTLGKLKDTPRTGWVENKIPNVESVADHMYRMSVLCMMCPD--TTLDKNRMIRMAL
XP_003722807 Leishmania major strain Friedlin              13 VISFLHTVGRLKVTARQGWVENQICSPESVSDHMYRMSLMCMMCPD--TSLNRDRMVKMAL
XP_821610    Trypanosoma cruzi strain CL Brener             5 TITFLRTVGKLKETDRTGWVEHGIPNPESVSDHMYRVAVMCMMCPD--EKLDRNKLIRMAL
XP_827362    Trypanosoma brucei brucei TREU927              3 TIEFLHTVGKLKETMRTGWVETGVHQPESVSDHMYRAALLCMMCPD--SSLNRDRLVRMAL
CCW68923     Phytomonas sp. isolate Hart1                  13 AISFLQTIGRLKDTPRTGWVENGIANAESVGDHMYRMALMCMMCPD--PSLDKTRMIQMAL

                                                            MM  C    R
SS                                                          H  HHHHHH          HHHHHHHHHHHHHH            HHHHHHHHHH   HHH
1xx7_A       Pyrococcus furiosus                            IHDLGEAIITDLPLS-AQKYLNKEEAEAKALKDVL------------PEYTELFEEYSKALTLEG
1ynb_A       Archaeoglobus fulgidus                         FHDLHEARTMDLHKIA---RRYVSCDEEGAREEQL-----------------SWMESKPDFSDVE
3kh1_A       Magnetospirillum magnetotacticum               IHDIVEIDAGDTFIHD---EADKEERERKAAARLFGLLPP----DQAAEYSALWQEYEARETADA
2pau_A       Escherichia coli K-12                          YHDASAVLTGDLPTP---IAQEYKAIEKIAQQKLVDMVPE----ELRDIFAPLIDEH-AYSDEEK

NP_057147    Homo sapiens                                   VHDMAECIVGDIAPADNIPKEEKHRREEEAMKQITQLLPE----DLRKELYELWEEYETQSSAEA
NP_001264847 Gallus gallus                                  VHDMAECIVGDIAPADNISKEEKHRREEAAMRQLTQLLSE----DLKKEIYELWEEYENQCTAEA
XP_002936040 Xenopus tropicalis                             VHDMAECIVGDIAPADNISKEEKHRKEKDAMQHLTQLLPD----ILKTEVYNLWEEYEHQSTAEA
NP_001038696 Danio rerio                                    VHDLAECIVGDIAPADNVSKAEKHRREKDAMVHITGLLDD----GLRKEIYNLWEEYETQSSPEA
NP_609052    Drosophila melanogaster                        VHDLAESLVGDITPFCGISKDDKRAMEFKAMEDICKLIE-----PRGKRIMELFEEYEHGQTAES
XP_306780    Anopheles gambiae str. PEST                    VHDLAEGIVGDITPYCGVSREEKLLKEFSAMTEIASLLG-----PNKDKMLALFNEYEEGKTPEA
NP_001256098 Caenorhabditis elegans                         VHDIGEAIAGDITPHCGVSDQDKFDLEKKAINTIASFVP-----NVGEEWTMLWKEYEEASSLTA
NP_587821    Schizosaccharomyces pombe 972h-                VHDMAESIVGDITPHENVSKEEKHRMESEAMVSITQQLIPLNLSLQAEEIKELFLEYESASTPEA
NP_009801    Saccharomyces cerevisiae S288c                 VHDIAESLVGDITPVDPIGKEEKHRREWETIKYLCNALIKPYNEIAAKEIMDDWLAYENVTSLEA
NP_973522    Arabidopsis thaliana                           VHDIAEAIVGDITPSCGISKEEKNRRESEALEHMCKLLGGG---ERAKEIAELWREYEENSSPEA
NP_001046081 Oryza sativa Japonica Group                    VHDIAEAIVGDITPSDGVPKEEKSRREQEALDHMCSLLGGG---PRAEEIRELWMEYEQNATLEA
CUG37041     Bodo saltans                                   CHDMAESIVGDISPGMKVPAEVKFERESTAMKHMTSLVPA----LGGEDMKGLWEEYEAQETAES
XP_003722807 Leishmania major strain Friedlin               CHDTGESIIGDISPAMKVPKEVKKQQESQAVQSLCKLVSSSPNTTFSKELGDLFEEYEAQETAES
XP_821610    Trypanosoma cruzi strain CL Brener             CHDAGESIVGDISPKMGVSKEDKYNQEKAAVLHLTGLLEKES--PLSRELHELWEEYEAQHTPEA
XP_827362    Trypanosoma brucei brucei TREU927              CHDVGESIIGDISPKMGVPAAEKHKREKEAILHLRGLLPHDS--PLEKELQELWEEYETQGTPEA
CCW68923     Phytomonas sp. isolate Hart1                   CHDAGESIIGDITPRSTITKEEKHVMELEAVHHLSELAFSAGCAPFSKDLSELFEEYEDQKTPES

                                                                  M
SS                                                          HHHHHHHHHHHHHHHHHH        HHHHHH       HHHHHHHHHHHHH
1xx7_A       Pyrococcus furiosus                            QLVKIADKLDMIIQAYEYELSGAK--NLSEFWNAL-EDLEKLEISRYLREIIEEVRRL--- 172
1ynb_A       Archaeoglobus fulgidus                         VYVSDADKLELAFQGVEYSQQVS---YAIRFAEN--VELKTDAAKEIYRVLMERKNPVWWR 167
3kh1_A       Magnetospirillum magnetotacticum               RFADALDRLQPLLHNFETE---GGTWKPHGVTR[7]PRIE-AGSKRLGAYARALVDEAVRR 191
2pau_A       Escherichia coli K-12                          SLVKQADALCAYLKCLEELAAGNN--EFLLAKT[6]EARRSQEMDYFMEIFVPSF------ 176

NP_057147    Homo sapiens                                   KFVKQLDQCEMILQASEYEDLEHKPGRLQDFYDSTAGKFNHPEIVQLVSELEAERSTNIAA 197
NP_001264847 Gallus gallus                                  KFVKQLDQCEMILQALEYEELENTPGRLQDFYDSTAGKFIHPEILQLVSLINTERNKKIAA 190
XP_002936040 Xenopus tropicalis                             KFVKELDQCEMILQALEYEELENRPGRLQDFYNSTAGKFNHPEVVQLVSAIYEERDSAIAA 194
NP_001038696 Danio rerio                                    KLVKELDNLEMIIQAHEYEELEGKPGRLQEFFVSTEGKFHHPEVLGLLKSLNEERARHIAA 183
NP_609052    Drosophila melanogaster                        KFVKDLDRLDMVMQAFEYEKRDNCLLKHQEFFDSTEGKFNHPFVKKLVNEIYEQRDVLAKA 359
XP_306780    Anopheles gambiae str. PEST                    KFVKDLDRLDMVMQAYEYEKRDSCPQKLQEFFDSTENKFSHPLVVDIVNAIKEQRAKAAST 184
NP_001256098 Caenorhabditis elegans                         RVVKHLDKFDMIVQADKYEKTHEI--DLQQFFTSTVGVLKMEPFATWDRELRENRMKRINK 185
NP_587821    Schizosaccharomyces pombe 972h-                KFVKDIDKFEMIAQMFEYERKFNGEKDLSQFTW-AGKLIQHPLVKGWLNDVLQEREQFWAS 192
NP_009801    Saccharomyces cerevisiae S288c                 RYVKDIDKYEMLVQCFEYEREYKGTKNFDDFFG-AVASIKTDEVKGWTSDLVVQRQKYFAD 231
NP_973522    Arabidopsis thaliana                           KVVKDFDKVELILQALEYEQDQGK--DLEEFFQSTAGKFQTNIGKAWASEIVSRRKQH--- 257
NP_001046081 Oryza sativa Japonica Group                    KVVKDFDKVEMILQALEYEKEQGL--DLEEFFQSTAGKFQTDVGKAWAAEVASRRK----- 228
CUG37041     Bodo saltans                                   HFVRDMDLLEMIVQAHHYE-ASAE-KDLSGFYK-SGDRIKHPWARQILETLKATSPAKLRA 187
XP_003722807 Leishmania major strain Friedlin               HFVKDMDLLEMVVQAHSYESANPG-KDLGSFFR-SGANIHHPWARAIYETLLETRPYLAYK 195
XP_821610    Trypanosoma cruzi strain CL Brener             QFLKDIDLLEMVAQAHAYELAHPK-KDLSSFFV-SGEKIKHPWARNIYETLLRTRSSEK-- 183
XP_827362    Trypanosoma brucei brucei TREU927              RFLRDIDLLEMVTQAHAYEKTHPE-LNFDSFYV-SGEKIKHPWVRSIYDNLVSTRPSRKS- 182
CCW68923     Phytomonas sp. isolate Hart1                   NFVRDMDILEMVIQADSYQALYPS-KNLRTFFE-SGEKIQHPWARAIFEKLKKNNPFLNL- 194
```
